# Potent and selective inhibition of SH3 domains with dirhodium metalloinhibitors†
†Electronic supplementary information (ESI) available: Experimental procedures, metallopeptide characterization, and assay details. See DOI: 10.1039/c5sc01602a



**DOI:** 10.1039/c5sc01602a

**Published:** 2015-06-03

**Authors:** Farrukh Vohidov, Sarah E. Knudsen, Paul G. Leonard, Jun Ohata, Michael J. Wheadon, Brian V. Popp, John E. Ladbury, Zachary T. Ball

**Affiliations:** a Department of Chemistry , Rice University , 6100 Main St. , Houston , Texas , USA . Email: zb1@rice.edu; b Department of Genomic Medicine , Core for Biomolecular Structure and Function , University of Texas , M.D. Anderson Cancer Center , Houston , Texas , USA; c Eugene Bennett Department of Chemistry , West Virginia University , 217 Clark Hall , Morgantown , West Virginia , USA; d Department of Molecular and Cellular Biology , University of Leeds , LS2 9JT , UK

## Abstract

Src-family kinases (SFKs) play important roles in human biology and are key drug targets as well. However, achieving selective inhibition of individual Src-family kinases is challenging due to the high similarity within the protein family. We describe rhodium(ii) conjugates that deliver both potent and selective inhibition of Src-family SH3 domains. Rhodium(ii) conjugates offer dramatic affinity enhancements due to interactions with specific and unique Lewis-basic histidine residues near the SH3 binding interface, allowing predictable, structure-guided inhibition of SH3 targets that are recalcitrant to traditional inhibitors. In one example, a simple metallopeptide binds the Lyn SH3 domain with 6 nM affinity and exhibits functional activation of Lyn kinase under biologically relevant concentrations (EC_50_ ∼ 200 nM).

## Abstract 2

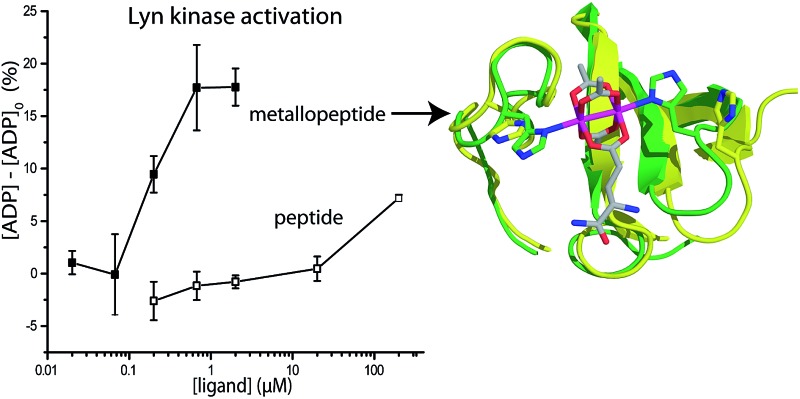
Specific, designed histidine–rhodium interactions allow a metallopeptide to bind Lyn kinase with nanomolar affinity and to activate kinase activity.

## Introduction

The Src-family SH3 domains are functionally important mediators of protein assembly and of signaling pathways that illustrate the problems of “undruggable” targets. SH3 domains are ubiquitous and versatile subunits, appearing ∼300 times in the human genome in proteins implicated in the proliferation of cancer and other diseases. The domains recognize short, proline-rich motifs (*e.g.* PxxP). However, our ability to chemically perturb Src-family SH3 interactions in a selective way is limited: SH3 domain interactions are weak (*K*_d_ ∼ 1–10 μM) interactions at a shallow binding interface and are highly conserved, especially among protein families such as the Src-family kinases. The Lyn kinase is a prototypical Src-family kinase. It contains a kinase domain and two regulatory domains: an SH3 and an SH2, which are believed to be involved in both upstream and downstream interactions.^1,2^ It shares significant sequence similarity with its Src-family brethren. Lyn and Lck, for example, have 63% sequence identity, similar to other comparisons within the family.

The SH3 domain represents an attractive and daunting challenge for inhibitor development. Within the Src family and in other related kinases, the catalytic kinase domain has been the primary target of inhibitor development. However, because the Src-family proteins have a high degree of similarity, kinase inhibitors can display unacceptable off-target activity. Thus the SH3 domain is a potentially powerful new target if truly selective inhibitors can be developed. In addition, SH3-selective inhibitors would shed light on kinase biology. The relative roles that Lyn SH2 and SH3 interactions play in the plethora of upstream and downstream signaling pathways known for Src-family kinases are poorly understood, apart from limited reports.^3,4^ The development of domain- and protein-specific tool compounds could untangle the roles SH3 domains play in kinase activation, catalytic reactivity, and substrate preference. Efforts to inhibit SH3 interactions have met with limited success, both in terms of potency and selectivity. Peptides^5,6^ and peptoids^7^ similar to the natural target sequence have been used in a variety of contexts to inhibit SH3 interactions, though IC_50_ ≥ 100 μM is typical. In one noteworthy approach, macromolecular, divalent ligands that bind simultaneously to SH3 and SH2 domains have been used to deliver increased potency.^8,9^ However, selectivity remains a general challenge when targeting Src-family kinases or members of other closely-related families.^10^ One small molecule, reported to disrupt SH3 interactions, was later shown to have no SH3 affinity.^11–13^ The Pyke group has reported 2-aminoquinolines that bind the Tec SH3 (∼10–100 μM), perhaps the most effective small molecule inhibitors to date.^14,15^ The HIV Nef protein binds tightly to the Hck SH3 domain, and exhibits half-maximal activation of Hck at 130 nM,^14^ though Nef also displays promiscuous activation of several other Src-family kinases.^16,17^

Dirhodium conjugates have unique properties that make them particularly well suited as inhibitors of specific protein–protein interactions. Dirhodium conjugates can benefit from metal–ligand interactions with histidine or other Lewis-basic residues on the surface of the target protein near the binding interface, offering potentially dramatic affinity benefits relative to traditional noncovalent organic interactions, which are typically weak. Dirhodium complexes are especially appealing in this regard due to a differential coordination environment—containing both exchange-inert equatorial sites that allow for stable conjugation and also weakly-held axial ligands for dynamic sampling of Lewis basic residues. Dirhodium complexes also have a history of biological and medical studies that indicate compatibility with living systems.^18–23^ The use of transition metals for such Lewis-basic anchoring of ligand molecules through specific interactions with the target protein is not widely studied. We have demonstrated inhibition with dirhodium cores in designed^24^ and natural protein^25,26^ contexts, and others have implemented similar ideas with cobalt^27,28^ and copper,^29^ for example. Designing metal coordination with protein target residues contrasts with alternative approaches that use transition metals as structural elements,^30,31^ oxidative damage agents,^32^ or cytotoxic species.^33,34^

## Results and discussion

**Fig. 1:**
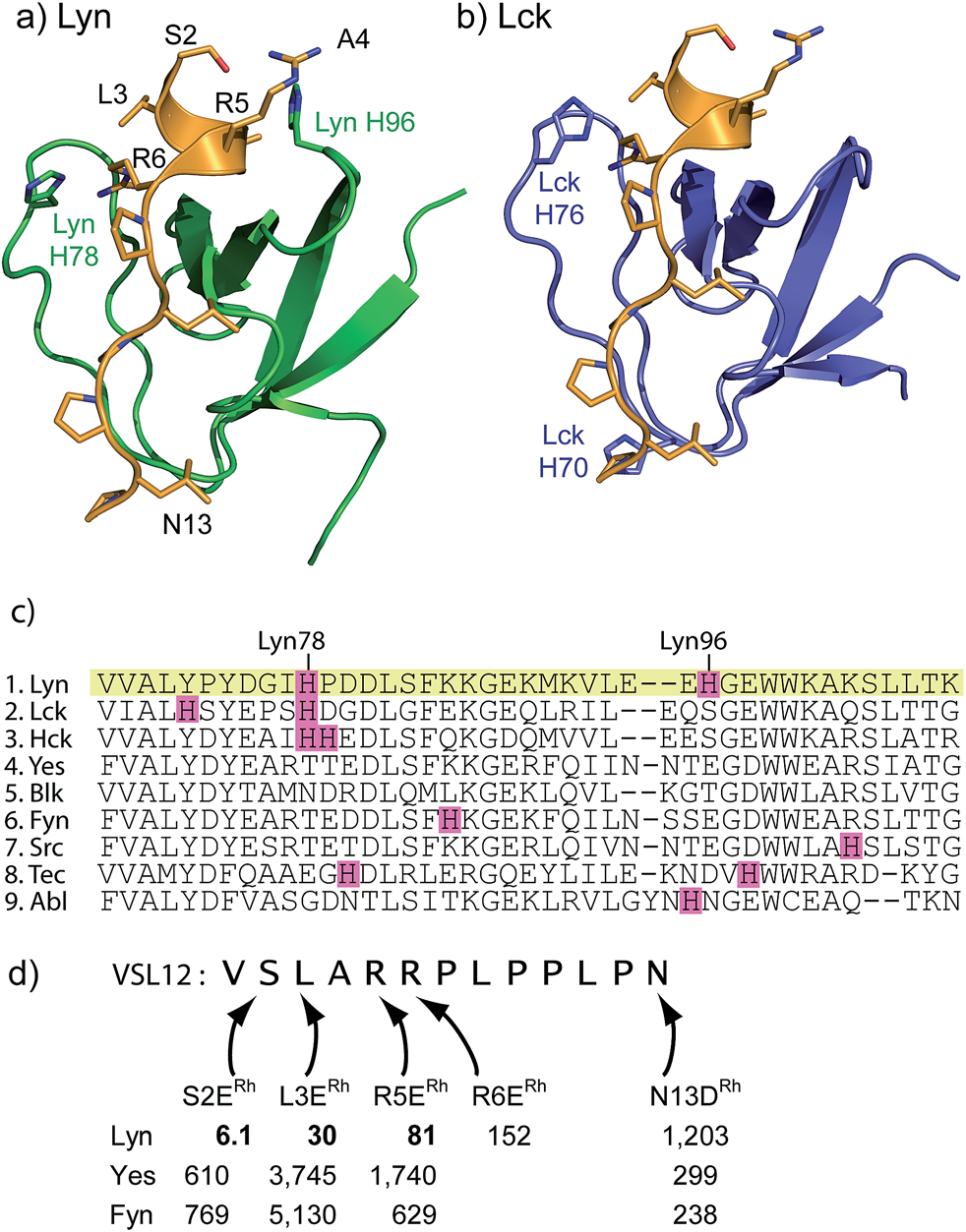
(a and b) Structures of the SH3 domains of representative Src-family kinases, Lyn (PDB ID: 1W1F)^35^ and Lck (PDB ID: ; 2IIM)^36^ with a peptide ligand (PDB ID: ; 4EIK). Histidine residues in the SH3 domain are shown explicitly. (c) Alignment of core SH3 residues for a variety of human Src-family (1–7) and other SH3 domains, highlighting histidine residues. (d) Affinity of designed rhodium(ii) metallopeptides for three Src-family SH3 domains.

Sequence and structure analysis shows that Lyn has two non-conserved histidine residues near the top of its binding pocket, His78 and His96 (Fig. 1a). The His96 is unique among Src-family sequences, and the His78 appears only in Hck and Lck. Individual amino-acid alterations are the only factors that might distinguish the nearly identical secondary structures and peptide-binding preferences of Src-family SH3 domains (*cf.* Fig. 1a and b). Based on a Src-family SH3-binding sequence (VSL12),^37^ known to bind with similar affinity across Src-family SH3 domains,^38^ we made metallopeptides with rhodium in different sites near the beginning of the SH3-binding peptide. Several of these showed increased affinity (Fig. 1). The optimal metallopeptide, S2E^Rh^, bound to Lyn with 6 nM affinity. The accuracy of ITC data is reduced for *C* values >1000 (*C* = [protein]/*K*_d_). Under these conditions the distribution of data points on the isotherm reduces the precision of the global fit. Since the enthalpic contributions to binding in the formation of the complexes described in this work were high, we were able to use a low protein concentration (4 μM) which ensured that our *C* values were well below this upper range. (The highest *C* value in our work is ∼670.) Isothermal titration calorimetry (ITC) measurements revealed an enthalpy-driven binding event (Δ*H* = –13.9 kcal mol^–1^) with a much smaller entropic penalty for binding (–*T*Δ*S* = 2.7 kcal mol^–1^) at 25 °C. In broad terms, the thermodynamics—favourable enthalpy and unfavourable entropy—are consistent with previous examinations of peptide–SH3 binding (see Table S1† for thermodynamic data).^39^ Moving the location of the rhodium center toward the N-terminus, away from the histidine, led to a drop in binding affinity. The N13D^Rh^ metallopeptide, a convenient negative control with the rhodium center too far for histidine interactions, binds with micromolar activity, similar to simple SH3 binding of the parent peptide. We conclude that the vast affinity improvement is due to rhodium coordination to unique histidine residue(s). The Hck domain—which shares one of two key histidine residues (Lyn His78; Hck His93) with Lyn—also exhibited significant affinity (*K*_d_ 26 nM, Table 1, entry 2) for the S2E^Rh^ metallopeptide.

**Table 1:** Affinity (*K*_d_) of selected metallopeptides for various human SH3 domains^*a*^

SH3 *K*_d_ (nM)								
Entry	Protein	S2E^Rh^	L3E^Rh^	R6E^Rh^	N13D^Rh^	P12D^Rh^	p40-A1E^Rh^	p40-Y4E^Rh^
1	Lyn	**6.1**	30	152	1203			
2	Hck	26^*b*^	52^*b*^	**51**	544	2315		
3	Lck	481		3788	239	**79**		
4	Yes	610	3745		301			
5	Fyn	769	5130		238			
6	Src	327						
7	Abl	5747			8850		**22**	7194

^*a*^All affinities measured by ITC.

^*b*^ITC measurements with Hck contain a second low-affinity (>20 μM) feature, presumably due to non-specific histidine interactions. Abl-binding peptide (p40) = APTYSPPPPP. p40-A1E^Rh^ = E^Rh^PTYSPPPPP. p40-Y4E^Rh^ = APTE^Rh^SPPPPP. Fyn binding data was previously reported (ref. 52).

The remarkable 6 nM affinity, far stronger than that of any reported non-protein ligand for SH3 domains, led us to study the structural basis of the observed affinity. Mutation experiments prove that both His78 and His96, in the Lyn SH3 binding pocket, contribute to potent binding. The H78A and H96A mutants bind to S2E^Rh^ with 20 and 39 nM affinity, respectively. The H78A and H96A double mutant binds much more weakly (>5 μM). These results indicate that *both* histidine residues bind to the dirhodium core, presumably by binding in a co-linear fashion to each of the two rhodium atoms. The two single-histidine mutants bind with roughly similar affinity, 3–7 fold less potent than the native Lyn, and ∼100× more potent than the parent peptide, implying that both histidines are well positioned to coordinate to the dirhodium center. That the majority of the stabilization energy comes from the first histidine coordination is consistent with the negative cooperativity generally seen for axial coordination to rhodium(ii): a second coordination in solution is roughly two orders of magnitude less favorable.^40–43^

The structure of the Lyn SH3 domain strongly suggests that two histidine residues are well positioned to coordinate to a dirhodium core, with the histidine side chains approaching from different directions and coordinating separate rhodium atoms. This bidentate trans coordination mode—reminiscent of metalloproteins such as cytochrome C^44^—is rare in small molecule ligands. We conducted a computational study using a combination of molecular mechanics and quantum mechanics to faithfully describe protein folding as well as rhodium coordination. Specifically, previously determined structures of Lyn (PDB ID: ; 1W1F) and Rh_2_(OAc)_4_ (ref. 45) were used as a starting model for the metalloprotein. Four structural isomers based on coordination of rhodium with His78 and His96 γ- and δ-nitrogen atoms were constructed. Geometry minimization was carried out on the four initial structures using the UFF force field in which the structural environment of Rh_2_(OAc)_4_ and the respective Rh–His bonds were frozen.^46^ The Glu carboxylate side chain of S2E replaced an equatorial acetate ligand in order to position the peptide near the SH3 binding groove. Two-layer ONIOM calculations were performed on Lyn–S2E^Rh^ isomers using the DFT functional B3LYP for the QM layer and the force field UFF for the MM layer. The most energetically stable isomer is predicted to arise from bis-histidine binding through both γ-nitrogen atoms.^47^ This binding motif also necessitates the least displacement of the native Lyn structure (Fig. S13†). In the optimized structure of the Nγ–Nγ isomer (Fig. 3a), the metallopeptide (yellow) overlays closely with a published structure for an SH3-binding peptide (magenta) in the C-terminal region. At the extreme N-terminus, on the other hand, the peptide backbone is displaced, and the short helical structure of the canonical peptide structure is replaced by the dirhodium core occupying the cleft between the two histidine residues (Fig. 3b). A slice depicting the histidine-containing region of the reported Lyn SH3 structure (Fig. 3c, yellow) and the calculated Lyn structure bound to S2E^Rh^ (green), demonstrates what little backbone alterations are needed to accommodate bis-histidine binding to the rhodium core. Based on the model, only small conformational changes in the Glu95–Trp99 and Ile77–Asp80 loops are required to position His78 and His96 to interact with the dirhodium tetraacetate, consistent with the small entropic penalty for binding observed in the ITC measurements.

**Fig. 2:**
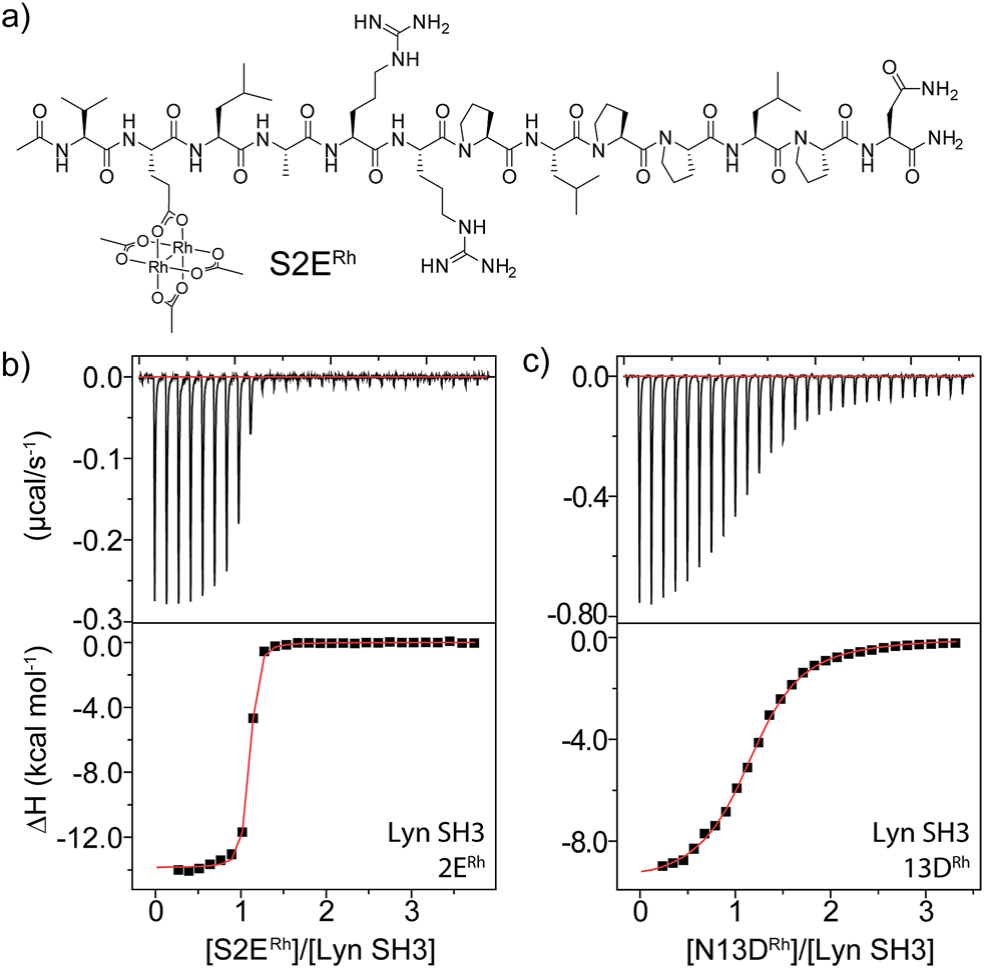
(a) Structure of the optimal Lyn-binding peptide, S2E^Rh^. (b and c) ITC analysis for affinity determination of S2E^Rh^ (b) and a negative control (c).

**Fig. 3:**
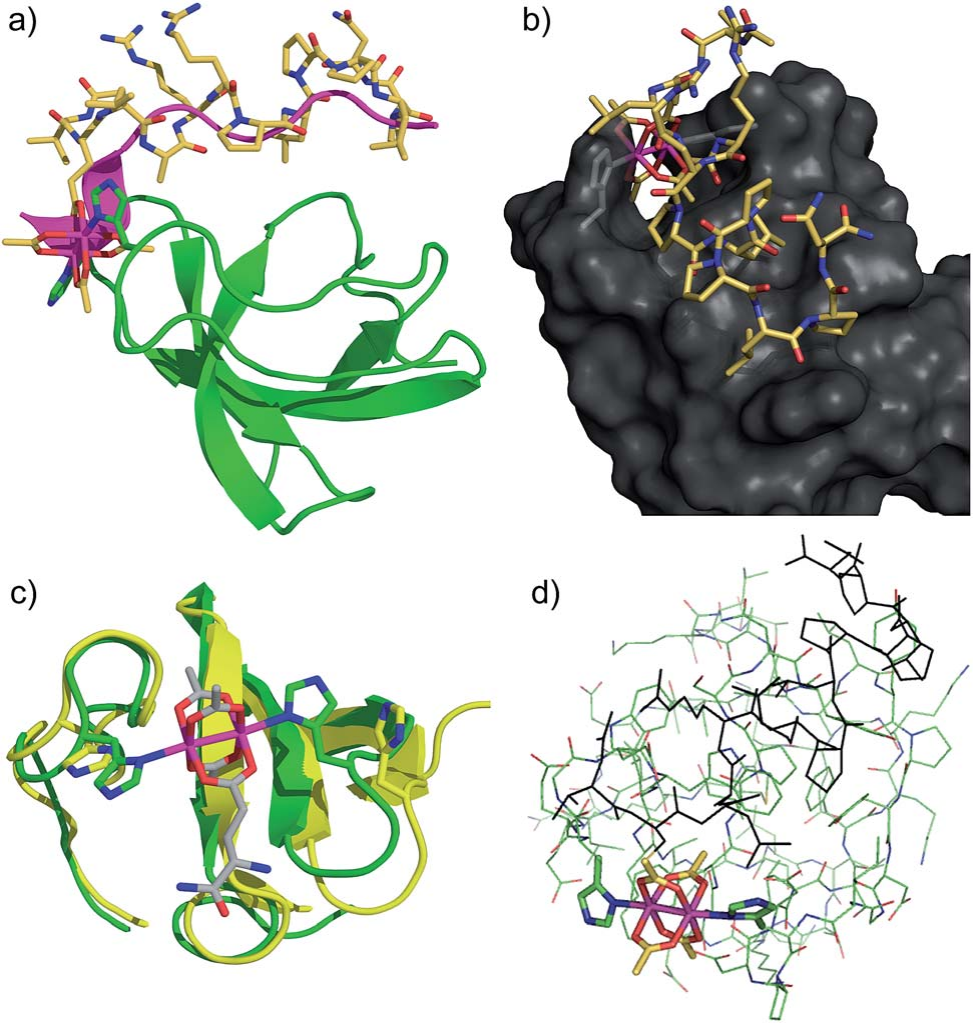
Computational models of S2E^Rh^ bound to Lyn SH3. (a) QM/MM-optimized structure of Nγ–Nγ isomer of Lyn–S2E^Rh^ with overlayed native SH3-binding peptide ligand (magenta). (b) Depiction of the histidine-flanked cleft of Lyn where dirhodium binding occurs. (c) Top slice view of an overlay of native Lyn SH3 (yellow) and the Nγ–Nγ isomer of Lyn–S2E^Rh^ (green). (d) Overview of the QM/MM optimization. High layer (DFT): tube. Low layer (MM): stick. See text and ESI† for details.

Rhodium(ii) conjugation represents a general way to build potent SH3 ligands in a predictable way from structural information and simple design principles. Through a combination of sequence optimization and judicious choice of rhodium location, it is possible to alter specificity to favor other SH3 domains. Lck is another Src-family protein, with high similarity to Lyn and possessing similarly SH3-binding-peptide preferences.^48^ However, Lck lacks the Lyn His96 residue (Fig. 1), and does not bind tightly to S2E^Rh^ (Fig. 2). On the other hand, Lck has a unique histidine (His70, see Fig. 1b) residue at the bottom of the pocket. By moving the rhodium core to the 12th residue (P12D^Rh^), the affinity for Lck increased to 79 nM as a consequence of specific interactions with the unique His70 residue (Fig. 2). Similarly, selective affinity for a third Src-family member, Hck, could be achieved with the R6E^Rh^ peptide (Table 1, entry 2), presumably due to interactions with the unique His94 (homologous to Lyn 79) found in Hck. The clean formation of 1 : 1 protein/metallopeptide complexes, even in the presence of excess metallopeptide was also indicated by analytical FPLC (see ESI†).

**Fig. 4:**
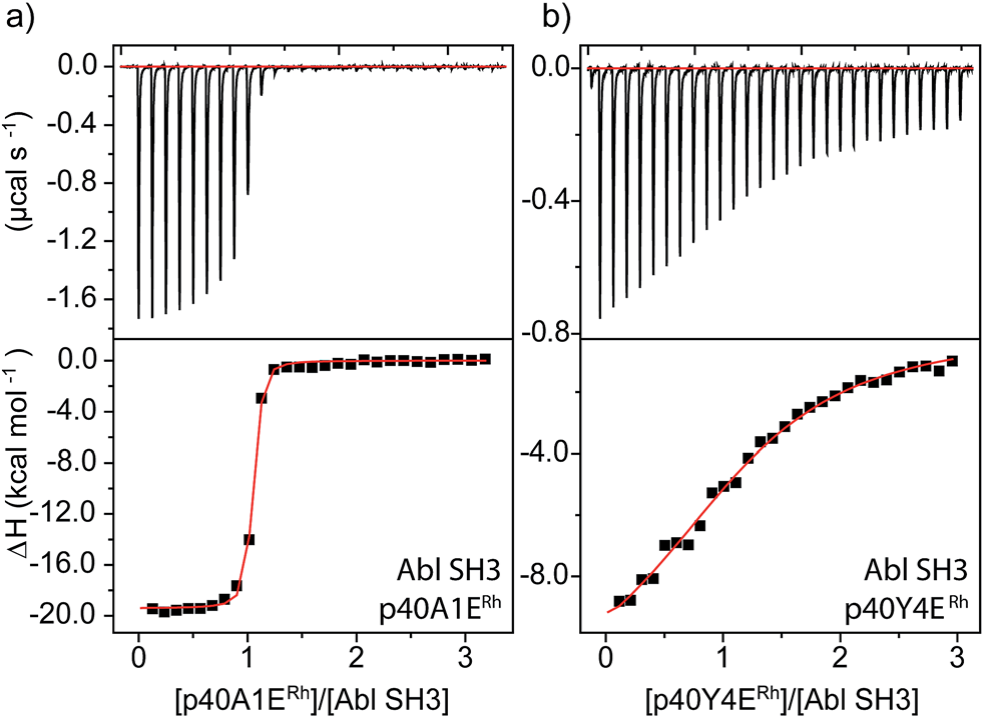
ITC analysis of Abl SH3 binding to designed metallopeptides.

An even more dramatic effect was observed with the Abl, a kinase outside of the Src family. The Abl kinase, and its constitutively active mutant variants, play key roles in tumor growth, and Abl is an important protein in cancer biology and a therapeutic target.^8,49,50^ Potent ligands for the Abl SH3 likewise represent an unmet need. Despite strong homology, Abl has a different peptide sequence preference than the Src-family kinases; it generally exhibits >5 μM affinity for peptides that interact with Src-family SH3 domains (Table 1). Abl binds the peptide p40 with 0.40 μM affinity.^51^ Abl also has a unique and accessible His95 residue (homologous to Lyn 95, Fig. 1). We designed two rhodium-containing variants of the Abl-binding peptide, p40-A1E^Rh^ and p40-Y4E^Rh^. While p40-Y4E^Rh^ did not improve binding, the p40-A1E^Rh^ peptide, which modeling studies suggested is better designed to position the rhodium core near the key His95 residue, binds Abl with 22 nM affinity, similar to our best Src-family ligands (Table 1 and Fig. 4). As with Lyn, the p40-A1E^Rh^ peptide represents the most potent ligand for the Abl SH3 domain yet reported.

**Fig. 5:**
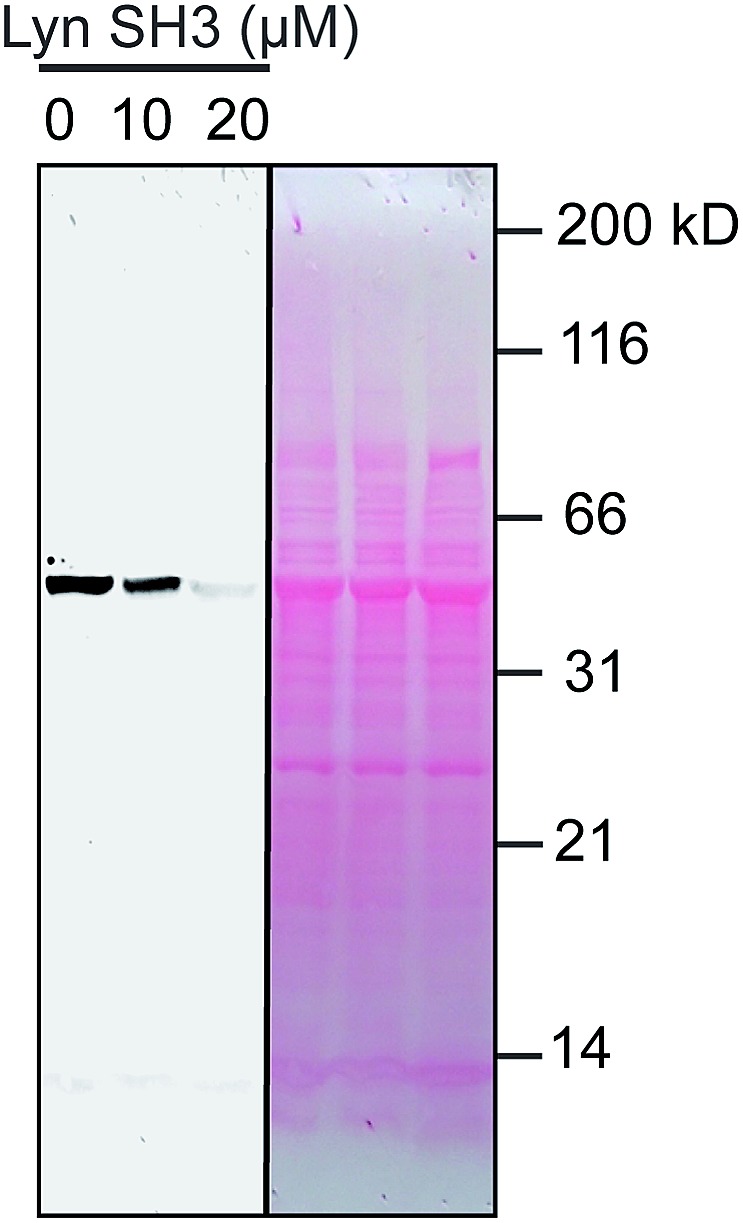
Potent sequestration of a metallopeptide catalyst by Lyn SH3. (left box) In the absence of Lyn, a metallopeptide (R5E^Rh^) catalyzes covalent attachment of an alkyne-containing small molecule to the Yes SH3 domain (expressed as a fusion with MBP) in cell lysate, visualized after reaction with a fluorogenic azide.^53^ The activity of the rhodium metallopeptide catalyst is inhibited by added Lyn SH3, indicating selective binding in lysate. (right box) Total protein (Ponceau) stain of the lysate reactions. Conditions: MBP–Yes fusion (2 μM), metallopeptide (10 μM), and MBP–Yes fusion (2 μM) in *E. coli* lysate, diluted 2× with *tert*-butylhydroxylamine buffer at pH 6.2 at 4 °C.

We used catalytic protein modification to examine the potency of the metallopeptide–Lyn interaction in a cell-like environment. We have previously shown that rhodium metallopeptides catalyze protein modification in lysate, with specificity provided by molecular recognition.^52^ More recent work has demonstrated that SH3 domains are amenable to this approach, permitting site-specific alkyne functionalization of specific SH3 domains in lysate.^53^ For example, in the presence of R5E^Rh^ metallopeptide and an alkyne–diazo reagent, the Yes SH3 domain (expressed as a fusion with maltose-binding protein, MBP) is readily tagged with an alkyne group, and the modification visualized by alkyne–azide cycloaddition on a blot membrane (Fig. 5, left box).^53^ Because catalytic covalent modification requires metallopeptide (R5E^Rh^) binding to the Yes SH3 domain, the addition of an exogenous high-affinity domain (Lyn, *K*_d_ = 81 nM) would be predicted to out-compete metallopeptide binding to the substrate (Yes, *K*_d_ = 1740 nM) and thus to prevent modification. Indeed, when these reactions are dosed with Lyn SH3, a drastic drop in labelling is observed, consistent with Lyn effectively outcompeting Yes and all other cellular proteins for the metallopeptide. Lyn itself is not modified by the catalyst, consistent with a Lyn–metallopeptide binding model (Fig. 3b) in which both rhodium coordination sites are blocked by histidine residues.

The potent Lyn SH3 binder we developed has functional consequences for Lyn kinase. The SH3 domain plays many regulatory and specificity roles for the Src family kinases *in vivo*. In full-length kinase, the SH3 domain is bound to a portion of the catalytic kinase domain, which abrogates kinase activity. The functional consequence of ligand binding to the SH3 domain is release of the SH3 domain from the kinase domain and subsequent full activation of kinase activity. Previous work indicates that SH3 interactions are necessary and sufficient for complete kinase activation, while SH2 interactions have a smaller effect.^5^

**Fig. 6:**
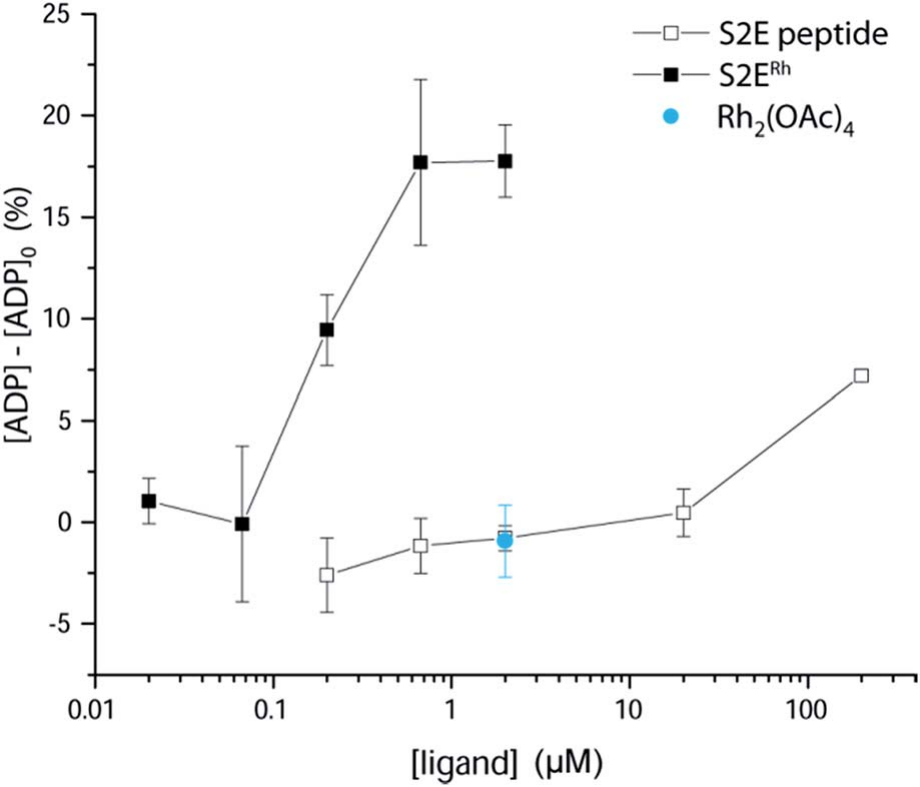
Activation of Lyn kinase activity by a metallopeptide, S2E^Rh^, and the parent peptide, S2E. The negative control, Rh_2_(OAc)_4_, had no effect on kinase activity. Full-length Lyn kinase (74 nM) was treated with substrate peptide and ATP. Kinase activity was measured after 5 min in an adaptation of reported methods.^5^ See ESI† for details.

We examined phosphorylation of a peptide substrate at low enzyme concentration (0.074 μM) and short reaction time (*t* = 5 min) to minimize the alternative autophosphorylation activation pathway (Fig. 6). Kinase activation with traditional ligands has typically required high ligand concentrations (∼1 mM) to overcome the intramolecular nature of the SH3–kinase domain interaction. Consistent with previous results, the parent peptide, S2E, showed slight activation only above 100 μM. Simple rhodium complexes (Rh_2_(OAc)_4_) displayed no activation (blue circle). However, the S2E^Rh^ metallopeptide exhibited strong Lyn activation at 200 nM, roughly three orders of magnitude lower than the parent peptide. Complete activation response was achieved at 630 nM. The only known SH3 ligand that exhibits comparable levels of activation within the Src family is the full-length HIV Nef protein, which binds tightly to Hck kinase. Taken together, the kinase activation and lysate-based inhibition demonstrate activation of the kinase target and activity in cell-like environments, and they provide confirmation of the potency gains made possible by rhodium conjugates.

## Conclusions

The S2E^Rh^ metallopeptide is the first ligand with single-digit nanomolar affinity yet reported for Lyn SH3 and is among the very few highly potent SH3 ligands yet reported. In addition, the S2E^Rh^ metallopeptide is the first reported small molecule that exhibits functional activation of Lyn kinase activity under biologically relevant concentrations. Importantly, an approach based on metallopeptides allows both structure-guided inhibitor design and selective inhibition within homologous protein families that are difficult to differentiate with traditional inhibitors. By targeting unique residues at the periphery of the binding pocket, we are able to design specificity for Lyn, and, separately, for Lck and Hck, despite the large sequence homology of Src-family SH3 domains. A nanomolar Abl ligand demonstrates that the approach can be generalized to SH3 types beyond the Src family. Rhodium-containing inhibitors thus should serve as powerful tools to probe homologous protein families.
