# Supplementary material for: Potent and selective inhibition of SH3 domains with dirhodium metalloinhibitors
Source: Chem Sci. 2015 Jun 3;6(8):4778–83. doi: 10.1039/c5sc01602a (PMC5667506; doi:10.1039/c5sc01602a)
Supplement: Supplementary file 1 [file SC-006-C5SC01602A-s001.pdf]

Electronic Supplementary Material (ESI) for Chemical Science.
This journal is © The Royal Society of Chemistry 2015

# Potent and selective inhibition of SH3 domains with dirhodium metalloinhibitors

Farrukh Vohidov[a], Sarah E. Knudsen[a], Paul G. Leonard[b], Jun Ohata[a], Michael J. Wheadon[a], Brian V. Popp[c], John E. Ladbury[b], and Zachary T. Ball[a]*

[a.] *Department of Chemistry, Rice University, Houston, Texas, United States*

[b.] *M.D. Anderson Cancer Center, The University of Texas, Houston, Texas, United States*

[c.] *Eugene Bennett Department of Chemistry, West Virginia University, Morgantown, West Virginia, United States*

*zb1@rice.edu

## Supplementary Information

## Table of contents

**General information** S2

**Experimental procedures** **S**4

**Supplementary figures** **S**5

HPLC and ESI-MS spectra of metallopeptides S5

ITC isotherms S13

Analytical FPLC spectra of metallopeptide - SH3 domain complex S18

Table of the thermodynamic parameters S19

Computational figures S20

**References** **S**22

## General information

All chemical reagents were purchased from Sigma-Aldrich and were used without further purification.

**Peptide synthesis.** All peptides were synthesized manually or with an AAPPTEC APEX 396 Automated Multipeptide Synthesizer using standard solid-phase Fmoc protocols.[1,2] Peptides were prepared as C-terminal amides using Rink amide MBHA resin (AAPPTEC) and were acetylated at the N-terminus prior to cleavage from the resin. Peptides and metallopeptides were purified by reverse-phase HPLC.

**HPLC separation.** Reverse-phase HPLC was performed on a Shimadzu CBM-20A instrument with Phenomenex Jupiter 4µ Proteo 90A (250 × 15 mm preparative) and Phenomenex Jupiter 4µ Proteo 90A (250 × 4.6 mm analytical) columns. The columns were eluted with a gradient of acetonitrile in water (10-90%) (flow rates of 8 mL/min and 1 mL/min for preparative and analytical columns, respectively). Trifluoroacetic acid (0.1%) was added to all eluents.

**FPLC separation.** Analytical FPLC was performed on an ÄKTA instrument with Superdex Peptide PE 7.5/300 column. The column was equilibrated with buffer (150 mM NaCl, 50 mM Tris, pH 7.0) at flow rate of 0.2 mL/min.

**ITC analysis.** ITC experiments were carried out on a VP-ITC instrument (MicroCal) at 25 ˚C. Titrations of 275 µM peptide into 18 µM SH3 domain proteins were used for all titrations, except for the titration of $S2E^{Rh}$ into Lyn SH3 domain, where the lower concentrations of 69 µM $S2E^{Rh}$ peptide and 4 µM Lyn SH3 were used to attain a c-value for the titration within the accepted limts for ITC experiments (1000 < c < 1).[3]

Heats of dilutions were measured independently for each experiment by titrating 275 µM peptide into buffer (20 mM phosphate buffer, pH 7, 150 mM NaCl, 1 mM EDTA). All experimental data were fitted using a one set of sites binding model in Origin software (MicroCal) except for the data with Hck SH3 domain. With Hck only, the non-peptide specific binding of dirhodium tetraacetate to Hck SH3 domain could not be treated as negligible, despite the very weak interaction (88 µM), due to the larger enthalpy contribution observed for non-specific binding to this SH3 domain, so the titrations of peptide into Hck were fitted using a 2 set of sites binding model where the stoichiometry for the second set of sites was fixed at 2.0.

**Kinase assay.** ATP turnover by Lyn was measured using the ADP-Glo™ Kinase Assay (Promega) according to the manufacturer's protocol. Briefly, final concentrations of 72 µM ATP, 74 nM Lyn (GST-tagged Lyn B), and 240 µM substrate peptide (all from Promega) were combined in buffer (40 mM Tris, 50 µM dithiothreitol, 2 mM $MnCl_2$ and 0.1 mg/mL BSA at pH 7.5) with a solution of the desired treatment (or control) in the same buffer for a final reaction volume of 5 µL. The kinase reaction was allowed to proceed at room temp. for 5 min from addition of ATP before the start of the ADP-Glo protocol halted the reaction. Chemiluminescence data were acquired on a Tecan Infinite M1000 microplate reader in white 384 well low volume plates (Corning Costar 3674) with an integration time of 250 ms per well. Each treatment condition and control was run in triplicate and the results averaged; data are reported as change in activity relative to the buffer control, and error bars represent one standard deviation.

**Mass spectrometry.** ESI-MS was performed on a Bruker Daltonics micrOTOF instrument.

**Known compounds and proteins**. Several metallopeptides ($S2E^{Rh}$, $L3E^{Rh}$, $R5E^{Rh}$, and $13D^{Rh}$) were reported previously and were prepared by known protocols;[4] alkyne-diazo 3,[5] 3-azido-7-hydroxycoumarin[6] were synthesized following reported procedures; *fyn*, *yes*, *lck*, and *src SH3* protein domains were prepared following published procedures.[5] *Abl*, *hck*, and *lyn SH3* domain plasmids were adapted from the previously reported genes;[7] the gene templates were obtained from Addgene, ID numbers: 23939, 23879, and 23905, respectively.

**Abl SH3 domain preparation.** The *abl SH3* domain was amplified by PCR using Phusion DNA polymerase (Finnzymes). The reactions were performed following manufacture's protocol with Phusion HF buffer and 5% DMSO. Primers were designed as follows: 5'-GGATCCGAAAATGACCCCAACCTTTTCGTTG-3' (BamHI site underlined) and a reverse primer, 5'-GAATTCTTAACTGTTGACTGGCGTGATGTAGTTG-3' (EcoRI site underlined). The resultant product was digested with BamHI and EcoRI and subcloned into BamHI-EcoRI sites of pGex-KTO. Confirmed PCR fidelity by sequencing.

**Hck SH3 domain preparation.** The *hck SH3* domain was amplified by PCR using Phusion DNA polymerase (Finnzymes). The reactions were performed following manufacture's protocol with Phusion HF buffer and 5% DMSO. Primers were designed as follows: 5'-GGATCCGGCTCTGAGGACATCATCGTGGTTG-3' (BamHI site underlined) and a reverse primer, 5'-GAATTCTTACAGAGAGTCAACGCGGGCGACATAG-3' (EcoRI site underlined). The resultant product was digested with BamHI and EcoRI and subcloned into BamHI-EcoRI sites of pGex-KTO. Confirmed PCR fidelity by sequencing.

**Lyn SH3 domain preparation.** The *lyn SH3* domain was amplified by PCR using Phusion DNA polymerase (Finnzymes). The reactions were performed following manufacturer's protocol with Phusion HF buffer and 5% DMSO. Primers were designed as follows: 5’-GGATCCGAGGAACAAGGAGACATTGTGG-3’ (BamHI site underlined) and a reverse primer, 5'-GAATTCTTAGAGTTTGGCCACATAGTTGCTG-3' (EcoRI site underlined). The resultant product was digested with BamHI and EcoRI and subcloned into BamHI-EcoRI sites of pGex-KTO. Confirmed PCR fidelity by sequencing.

**Lyn SH3 mutagenesis.** Single point mutations His17→Ala and His35→Ala; double mutation His17→Ala, His35→Ala of Lyn SH3 were performed using New England Biolabs Q5 mutagenesis kit. The pGex-KTO *lyn SH3* construct was used as template. The mutations were performed following manufacture's protocol. Primers were designed as follows: for H78A mutant: 5'-TGATGGCATCGCGCCGGACGACTTGTCTTTC-3' and a reverse primer, 5'-TAGGGGTACAAGGCTACC-3 (mutation site underlined); for H96A mutant: 5'-CCTGGAGGAGGCGGGAGAATGGTGGAAAG-3' and a reverse primer, 5'-ACTTTCATCTTCTCTCCTTTC-3' (mutation site underlined). The double mutant was prepared sequentially, using the same mutation primers. Confirmed PCR fidelity by sequencing.

After transformation of each plasmid into *E. coli* BL21 (DE3) the resulting cells were cultured under standard conditions. Cells were grown at 37 °C to $OD_{600}$ of ~0.6 with 100 µg/mL ampicillin, at which point they were induced with 0.5 mM IPTG. Cells were further incubated at 18 ˚C for 16 h before harvesting. Cells were pelleted by centrifugation at 10,000 *g* for 10 min. The pellet was resuspended in 50 mM Tris (pH 7.5), 200 mM NaCl, 200-µg/mL lysozyme, 4 unit/mL DNase I, and 1 mM $MgCl_2$ and incubated on ice for 1 h., followed by sonication 4 × 30 s. Cellular debris was removed by centrifugation (17,000 g at 4 ˚C for 1 h). Supernatant was subjected to affinity column (Glutathione - agarose resin), washed with 10 column volumes of 20 mM Tris and 200 mM NaCl at pH 7.5**.** Bound protein was eluted with 20 mM Tris, 200 mM NaCl**,** and 10 mM reduced glutathione at pH 7.5. The protein was concentrated and the buffer exchanged into cleavage buffer (200 mM Tris-HCl, 1.5 M NaCl, 25 mM $CaCl_2$ at pH 8.4) using Amicon Ultra centrifugal filters. The GST-tag was cleaved by incubation with thrombin (4 unit/mL) at 4 ˚C overnight.

The cleaved affinity tag was removed by size exclusion chromatography on a Superdex 75 column (GE Healthcare) equilibrated in 20 mM phosphate buffer containing 150 mM NaCl and 1 mM EDTA at pH 7. The purity of eluted fractions was assessed by SDS-PAGE using 12% Bis-Tris gels (Invitrogen). Fractions containing pure SH3 domain protein were pooled, concentrated, and aliquoted for storage at –80 ˚C. Concentration was determined using the absorbance at 280 nm (NanoDrop, Thermo Scientific).

**Computational Methods:**

Four isomeric structures of $Rh_2OAc_4$ bound to the Lyn-SH3 domain (1W1F PDB) were obtained by molecular mechanics (MM) geometry minimization using the UFF force field as implemented within *Spartan '14*. The atoms of $Rh_2OAc_4$ and the rhodium-$N^{His}$ bonds were frozen during the calculation, allowing the protein to relax around the dirhodium center. The S2E peptide was subjected to a molecular dynamics simulation (Monte Carlo, parameters: $T_{initial}$ = 5000K and step size = 10 K) with *Spartan '14*, and the lowest energy conformation was added to the dirhodium center by substitution of the S2E glutamate side chain with that of an equatorial acetate ligand. QM/MM geometry optimization of the four resulting isomers was carried out using the ONIOM algorithm within *Gaussian03* (G03).[8] Two layer ONIOM calculations were carried out in which the high and low layers were calculated using density functional theory (DFT) and MM, respectively. The molecular makeup of the high layer is illustrated in Figure X of the manuscript. DFT calculations used the B3LYP functional[9] with the LANL2TZ(f) basis set for Rh, obtained from the EMSL Basis Set Library,[10] and the 6-31G(d) basis set for all other atoms. Smaller basis sets for Rh (eg., LANL2DZ) failed to reproduce the Rh-Rh bond length with acceptable computational accuracy (ie, 2.39±0.05 Å when imidazole-derived axial ligands were present).[11] MM calculations used the UFF force field as implemented within G03, and charges were estimated at the initial geometry using the QEq algorithm. Geometry optimizations were carried out using the "quadmacro" option in order to produce finer optimization steps. All calculations minimized to geometries that exceeded the default optimization criteria, but subsequent analytical frequency calculations were not performed due to the size of the systems. The four minimized isomers are depicted in Figure S-1. The Lyn-$Rh^{S2E}$ isomers' SH3 domains were each compared to that of the native Lyn-SH3 domain (972 atoms) using the "fit" algorithm within MacPyMol.[12] The structural similarity was characterized by the root-mean-squared (RMS) error and is tabulated in Figure S-2.

## Experimental procedures

**Representative peptide metalation for the preparation of rhodium metallopeptides: Preparation of metallopeptide $S2E^{Rh}$.** Purified S2E peptide (AcVELARRPLPPLPN-$NH_2$) (4.63 mg, 3.06 µmol) and $Rh_2(tfa)(OAc)_3$ (1.52 mg, 3.07 µmol) were added to a 4-mL scintillation vial with pH 4.7 MES buffer (1 mL, 0.1 M, MES=2-(*N*-morpholino)ethanesulfonic acid). The reaction was stirred with heating to 50 °C overnight, after which all reactants were consumed based on HPLC analysis (monitored at 220 and 300 nm). The resulting metallopeptide was purified by direct injection of the reaction mixture onto a preparative HPLC column and isolated by lyophilization to afford a light blue solid (4.27 mg, 73%). Purity was assessed by ESI–MS (calc'd for $[M+2H]^{2+}$ = 948.4, found 948.1) and analytical HPLC.

**In-lysate reaction conditions.** *Parallel MBP-Yes SH3 modification reactions in lysate with increasing amounts of added Lyn SH3*. Lysate (25 µL) from D3E *E. coli.* that had no transformed plasmid was buffered to pH 6.2 with *tert*-butyl-hydroxylamine·HCl (TBHA, 23 µL, 100 mM). Pure MBP-Yes SH3 fusion protein (2 µM final concentration), Lyn SH3 (0 – 10 µM final concentration), metallopeptide catalyst (0.5 µL, 10 µM final concentration), and alkyne-diazo **3** (0.5 µL, 500 µM final concentration) were added. Reactions were run for 16 h at 4 °C.

**Fluorogenic analysis of blot membranes.** In-lysate reaction sample (5 µL) was directly loaded into SDS-PAGE (12% Bis-tris gel, Life Technologies). Resolved protein bands were transferred onto a PVDF membrane (GE Healthcare), which was preactivated in MeOH for 5 mins. After transfer the membrane was bathed in a click reaction mixture containing 500 µM 3-azido-7-hydroxycoumarin, 20 µM Tris[(1-benzyl-1H-1,2,3-triazol-4-yl)methyl]amine (TBTA), 5 mM sodium ascorbate, 500 µM $CuSO_4$. The solvent for reaction was 50% DMSO/$H_2O$. After 1 h incubation with gentle rocking in the dark at room temp., the membrane was rinsed 3 times with 70% EtOH to remove DMSO. The membrane was imaged with a Fujifilm LAS-4000 instrument using epi-UV light source (370 nm LED) and L41 filter set. After fluorescence imaging, the membrane was air dried and then stained with Ponceau S solution for imaging of total protein loading.

**Supplementary figures**

**Characterization of metallopeptides**

**a)**

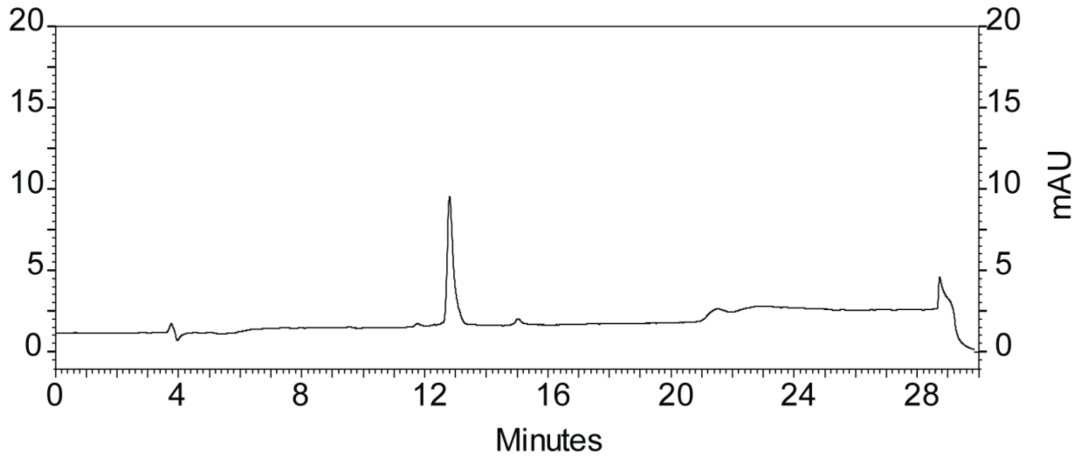


**b)**

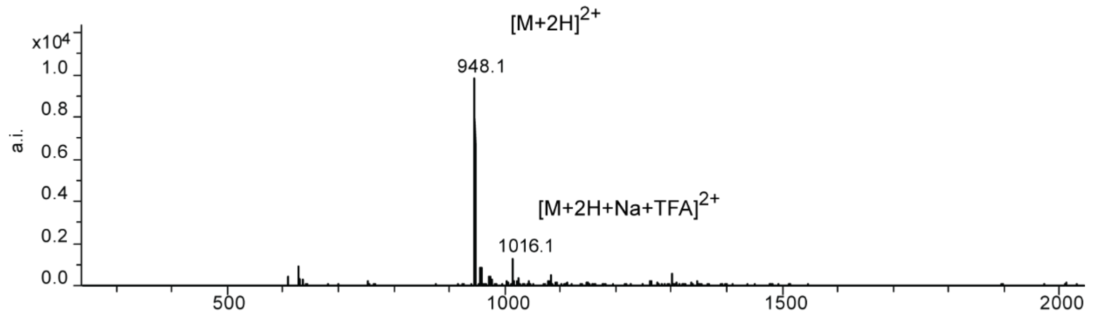


**Figure S1. a)** HPLC trace of **S2E$^{Rh}$** (0~1.0 min, $CH_3CN$ 10%, 1.0~17.0 min, $CH_3CN$ 10%~60%, 18.0-25.0 $CH_3CN$ 90%, 25.1-30.0 $CH_3CN$ 10%). **b)** ESI: calc'd mass 1894.73; $[M+2H]^{2+}$ 948.4; found 948.1.

**a)**

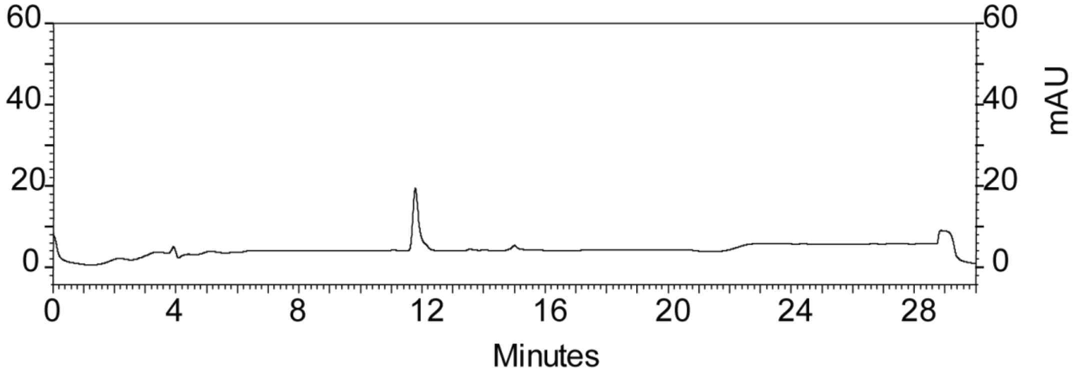


**b)**

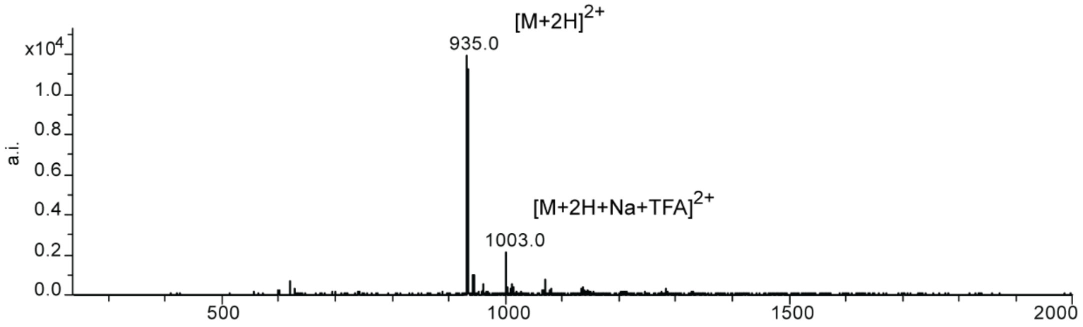


**Figure S2. a)** HPLC trace of **L3E$^{Rh}$** (0~1.0 min, $CH_3CN$ 10%, 1.0~17.0 min, $CH_3CN$ 10%~60%, 18.0-25.0 $CH_3CN$ 90%, 25.1-30.0 $CH_3CN$ 10%) **b)** ESI: calc'd mass 1868.68, $[M+2H]^{2+}$ = 935.4; found 935.0.

**a)**

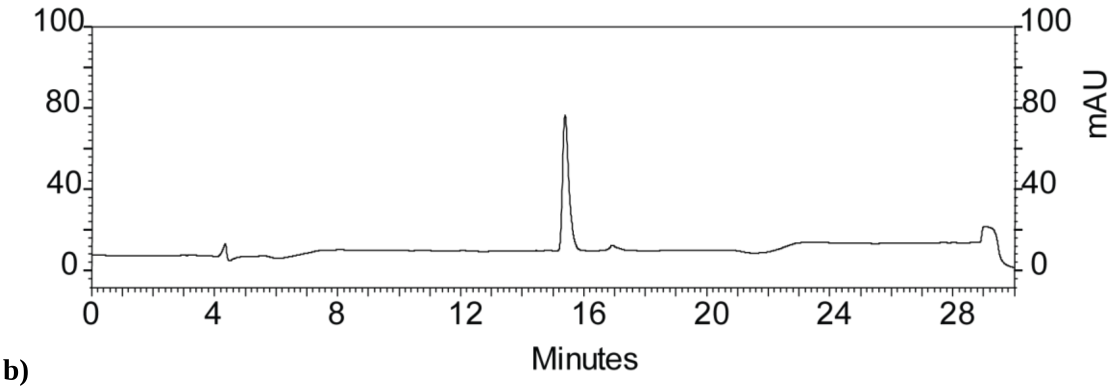


**b)**

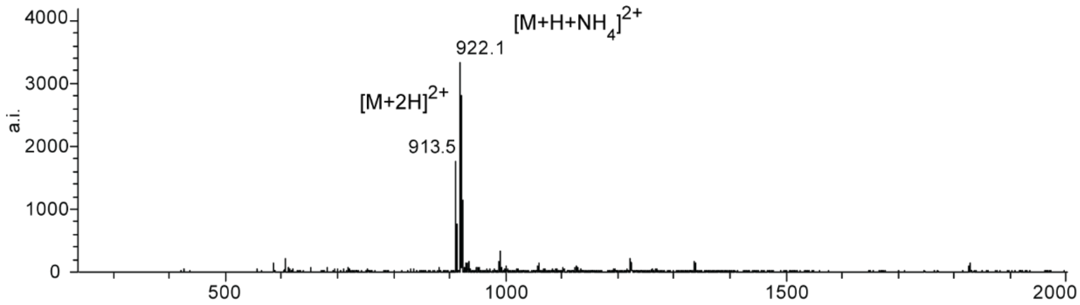


**Figure S3. a)** HPLC trace of **R5E$^{Rh}$** (0~1.0 min, $CH_3CN$ 10%, 1.0~17.0 min, $CH_3CN$ 10%~60%, 18.0-25.0 $CH_3CN$ 90%, 25.1-30.0 $CH_3CN$ 10%) **b)** ESI: calc'd mass 1825.66, $[M+2H]^{2+}$ = 913.8; found 913.5.

**a)**

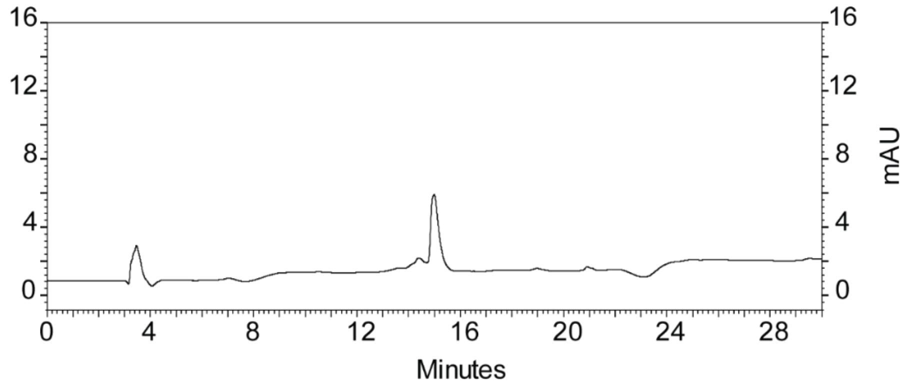


**b)**

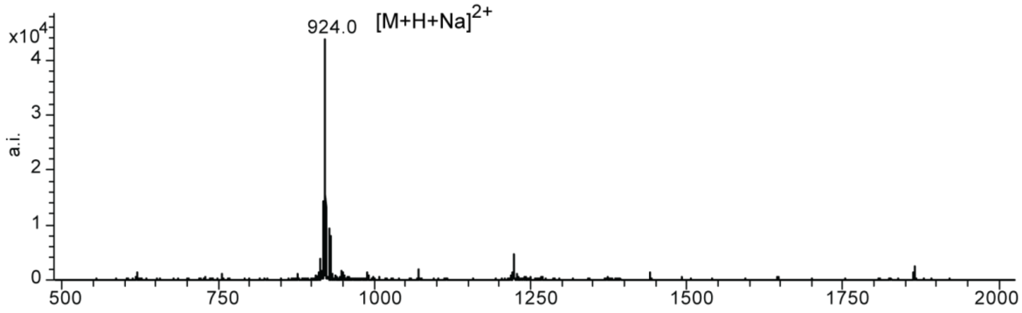


**Figure S4**. **a)** HPLC trace of **R6E$^{Rh}$** (0~1.0 min, $CH_3CN$ 10%, 1.0~17.0 min, $CH_3CN$ 10%~60%, 18.0-25.0 $CH_3CN$ 90%, 25.1-30.0 $CH_3CN$ 10%) **b)** ESI: calc'd mass 1825.66, $[M+H+Na]^{2+}$ 924.8; found 924.0.

**a)**

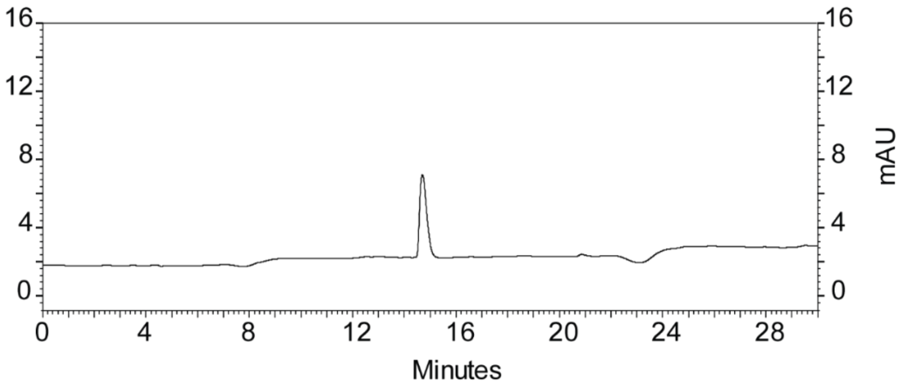


**b)**

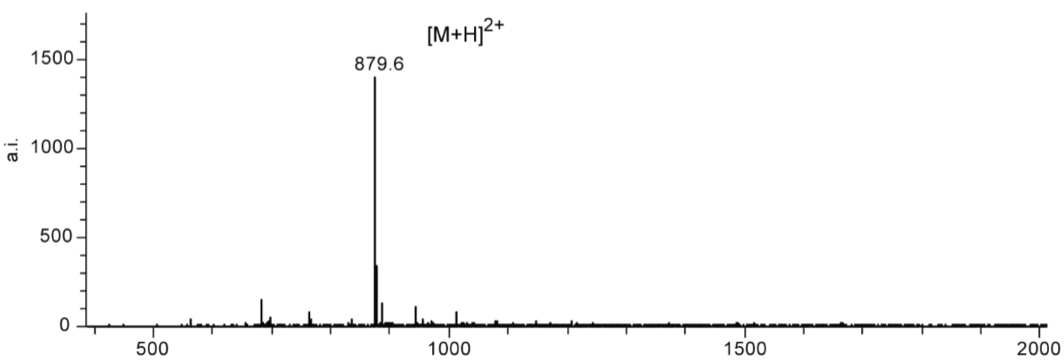


**Figure S5**. **a)** HPLC trace of **P12D$^{Rh}$** (0~1.0 min, $CH_3CN$ 10%, 1.0~17.0 min, $CH_3CN$ 10%~60%, 18.0-25.0 $CH_3CN$ 90%, 25.1-30.0 $CH_3CN$ 10%) **b)** ESI: calc'd mass 1756.65, $[M+H]^{2+}$ = 879.3; found 879.6.

**a)**

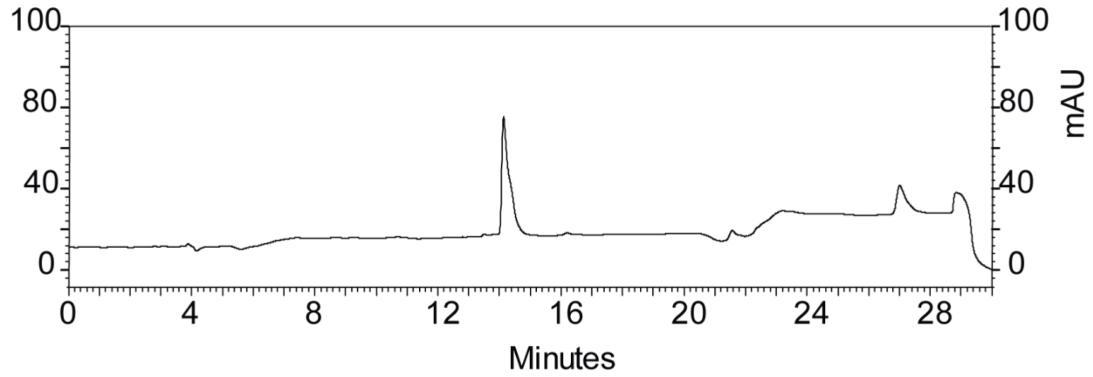


**b)**

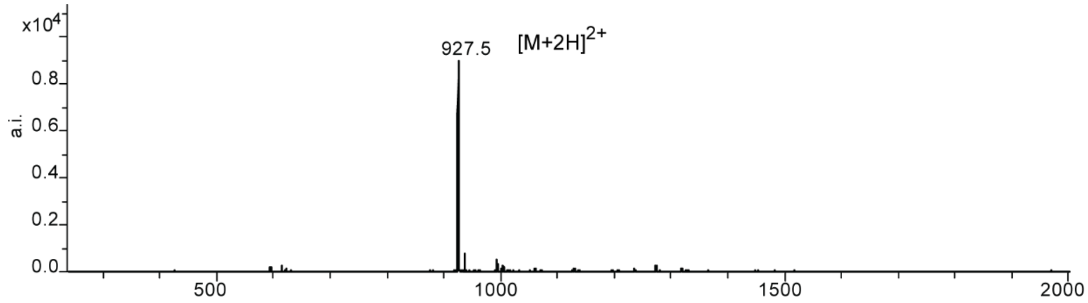


**Figure S6. a)** HPLC trace of **$13D^{Rh}$** (0~1.0 min, $CH_3CN$ 10%, 1.0~17.0 min, $CH_3CN$ 10%~60%, 18.0-25.0 $CH_3CN$ 90%, 25.1-30.0 $CH_3CN$ 10%) **b)** ESI: calc'd mass 1853.71, $[M+2H]^{2+}$ = 927.9; found 927.5.

**a)**

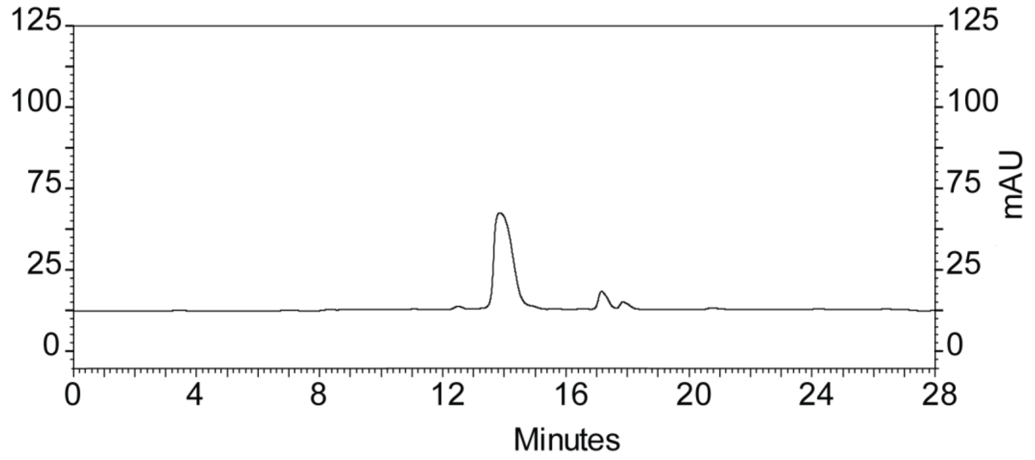


**b)**

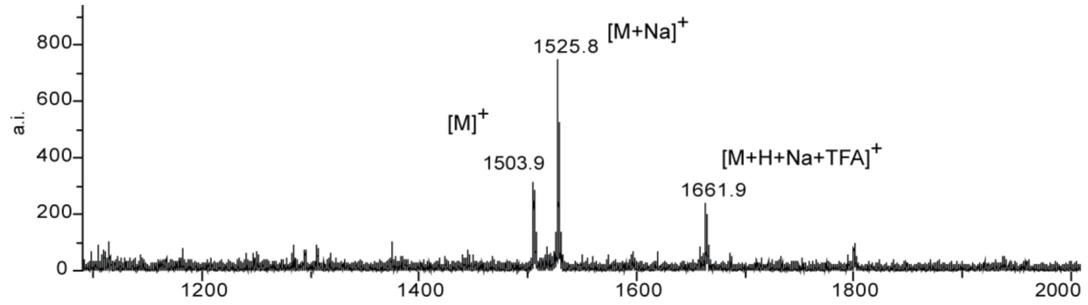


**Figure S7**. **a)** HPLC trace of **P40-A1E$^{Rh}$** (0~1.0 min, $CH_3CN$ 10%, 1.0~17.0 min, $CH_3CN$ 10%~60%, 18.0-25.0 $CH_3CN$ 90%, 25.1-30.0 $CH_3CN$ 10%) **b)** ESI: calc'd mass 1504.38, $[M]^+$ = 1504.4; found 1503.9.

a)

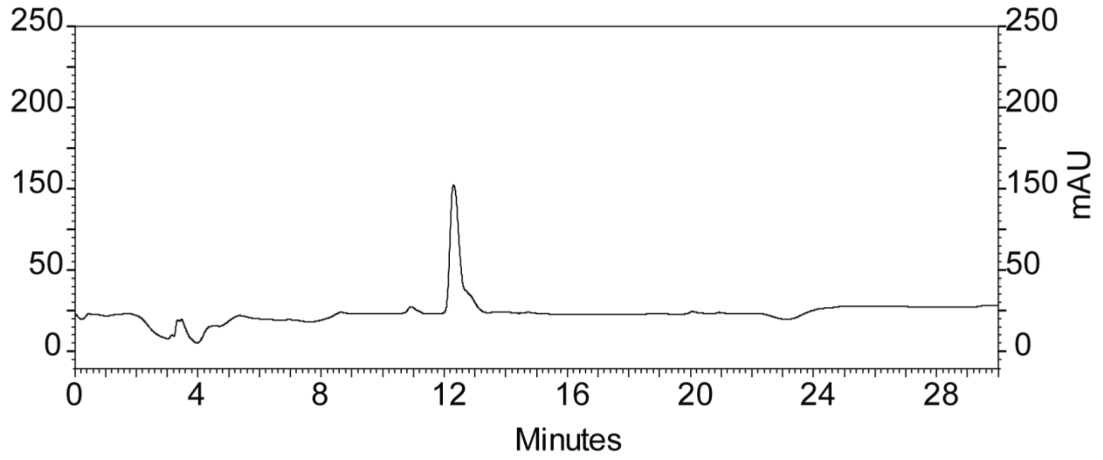


b)

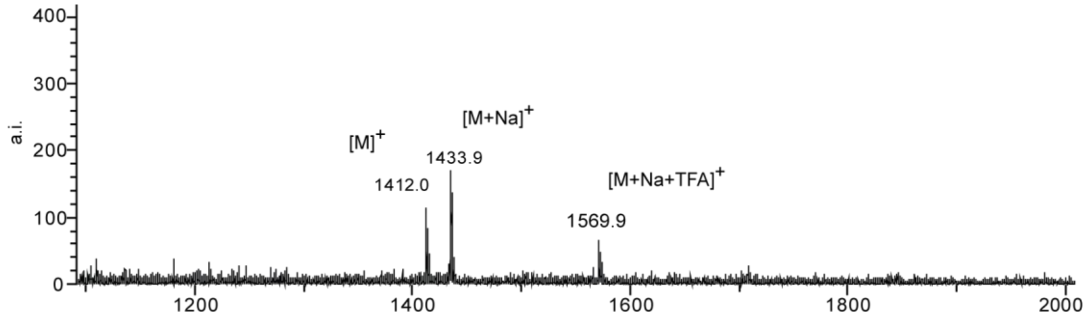


**Figure S8**. **a)** HPLC trace of **P40-Y4E$^{Rh}$** (0~1.0 min, $CH_3CN$ 10%, 1.0~17.0 min, $CH_3CN$ 10%~60%, 18.0-25.0 $CH_3CN$ 90%, 25.1-30.0 $CH_3CN$ 10%) **b)** ESI: calc'd mass 1412.35, $[M]^+$ 1412.4; found 1412.0.

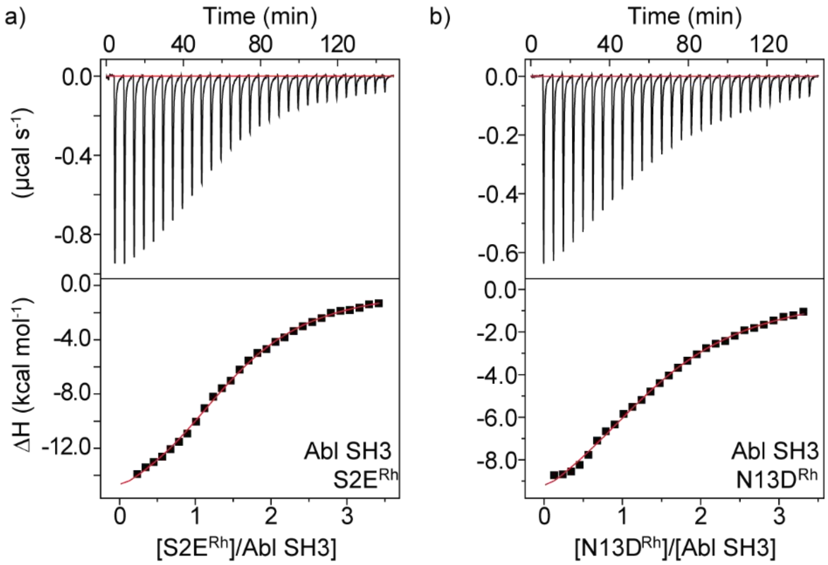


**Figure S9**. ITC titration of 275 μM metallopeptide into 18 μM Abl SH3 domain protein. a) $\mathbf{S2E^{Rh}}$, b) $\mathbf{N13D^{Rh}}$.

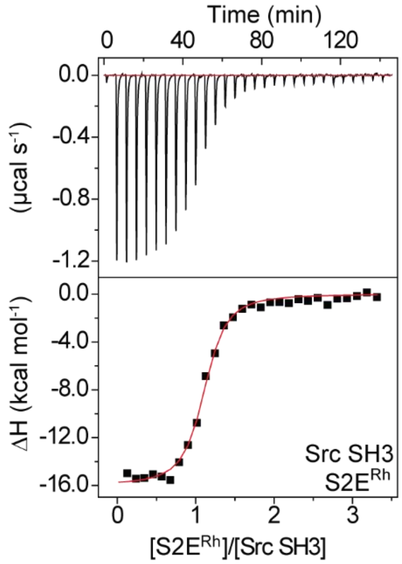


**Figure S10**. ITC titration of 275 μM $\mathbf{S2E^{Rh}}$ metallopeptide into 18 μM Src SH3 domain protein.

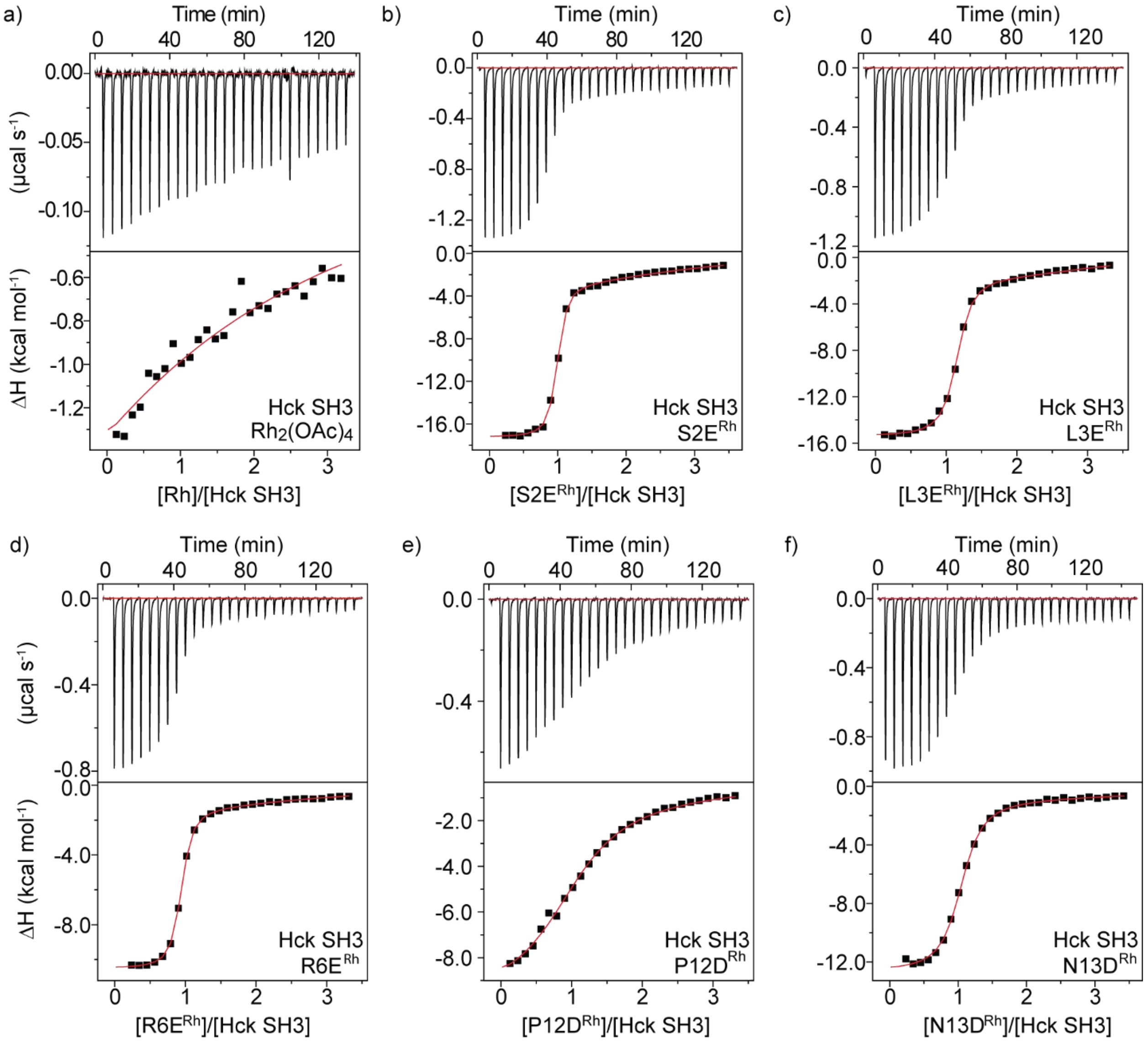


**Figure S11**. ITC titration of 275 µM metallopeptide or $Rh_2(OAc)_4$ into 18 µM Hck SH3 domain protein. a) $Rh_2(OAc)_4$, b) **S2E$^{Rh}$**, c) **L3E$^{Rh}$**, d) **R6E$^{Rh}$**, e) **P12D$^{Rh}$**, f) **N13D$^{Rh}$**.

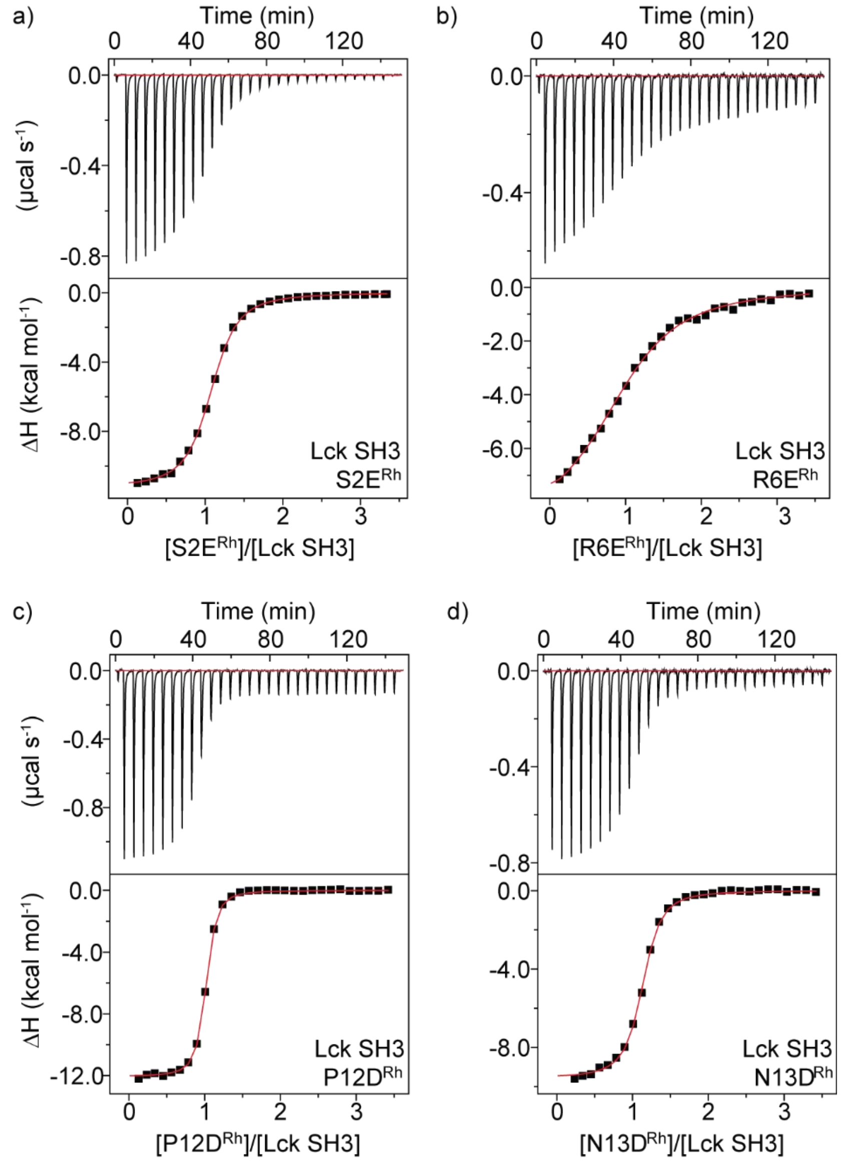


**Figure S12**. ITC titration of 275 μM metallopeptide into 18 μM Lck SH3 domain protein. a) $\mathbf{S2E^{Rh}}$, b) $\mathbf{R6E^{Rh}}$, c) $\mathbf{P12D^{Rh}}$, d) $\mathbf{N13D^{Rh}}$.

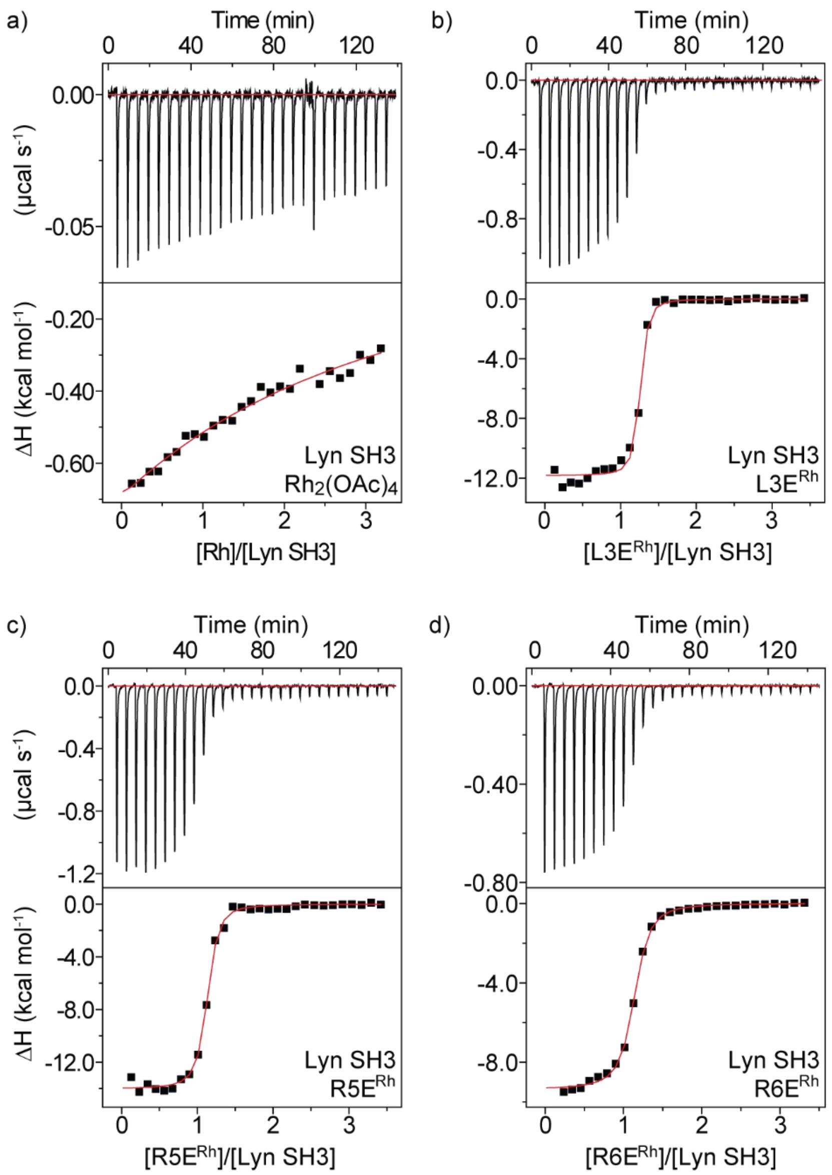


**Figure S13**. ITC titration of 275 µM metallopeptide or $Rh_2(OAc)_4$ into 18 µM Lyn SH3 domain protein. a) $Rh_2(OAc)_4$, b) $\mathbf{L3E^{Rh}}$, c) $\mathbf{R5E^{Rh}}$, d) $\mathbf{R6E^{Rh}}$.

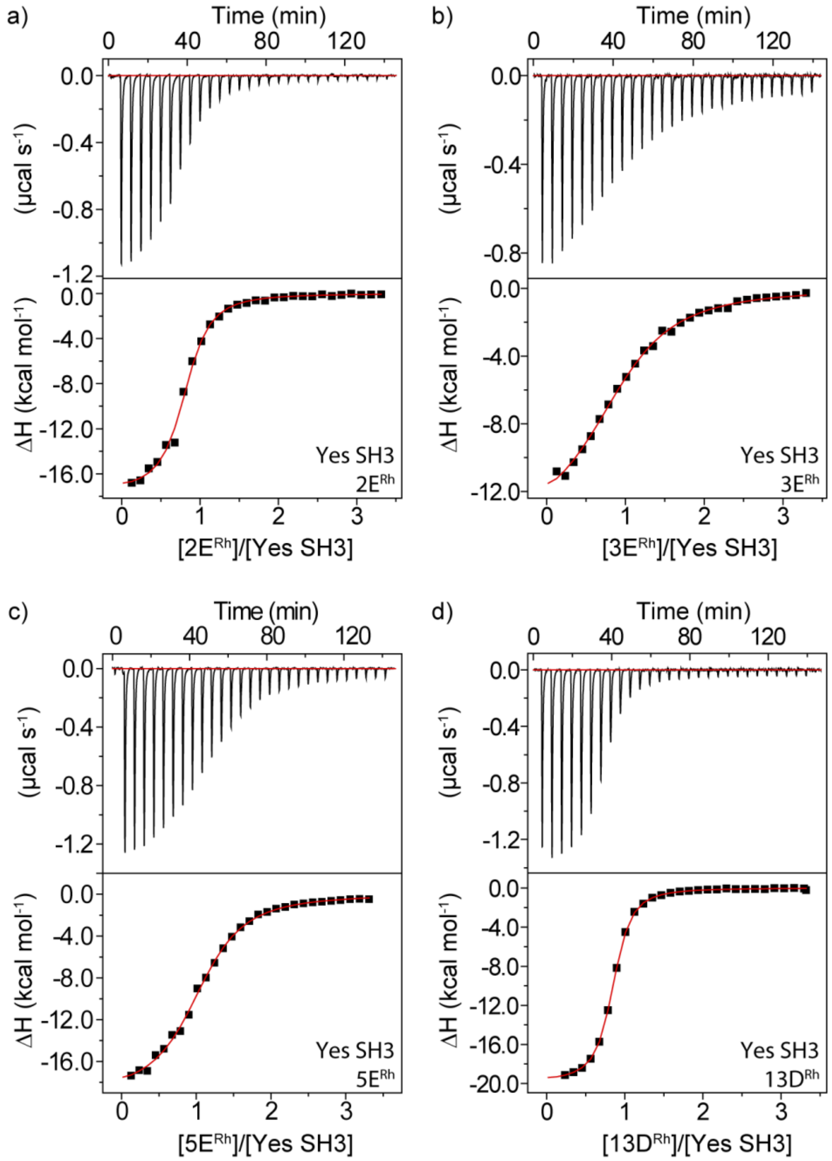


**Figure S14**. ITC titration of 275 µM metallopeptide into 18 µM Yes SH3 domain protein. a) **S2E**$^{\mathbf{Rh}}$, b) **L3E**$^{\mathbf{Rh}}$, c) **R5E**$^{\mathbf{Rh}}$, d) **N13D**$^{\mathbf{Rh}}$.

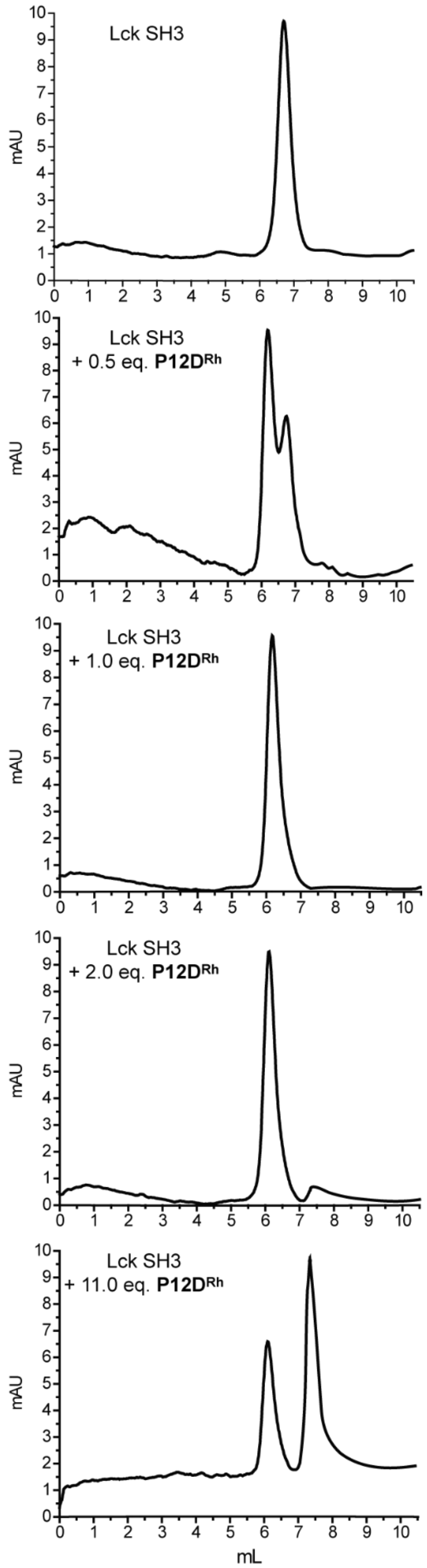


**Figure S15**. Analytical FPLC spectra of Lck SH3 complex with increasing amounts of **P12D$^{Rh}$** metallopeptide.

**Table S1.** Table of the thermodynamic parameters for binding of metallopeptides to SH3 domains, determined by isothermal titration calorimetry.

| Protein | Peptide | Stoichiometry | $K_d$ (nM) | ΔH (kcal $mol^{-1}$) | -TΔS (kcal $mol^{-1}$) | ΔG (kcal $mol^{-1}$) |
|---|---|---|---|---|---|---|
| Lyn | $Rh_2(OAc)_4$ | 1.0 (fixed) | 150000 | -6.3 | 1.0 | -5.2 |
| | $S2E^{Rh}$ | 1.1 | 6.1 | -13.9 | 2.7 | -11.2 |
| | $L3E^{Rh}$ | 1.2 | 30 | -11.8 | 1.6 | -10.3 |
| | $R5E^{Rh}$ | 1.1 | 81 | -14.0 | 4.4 | -9.7 |
| | $R6E^{Rh}$ | 1.1 | 152 | -9.4 | 0.1 | -9.3 |
| | $N13D^{Rh}$ | 1.2 | 1203 | -9.7 | 1.6 | -8.1 |
| Yes | $S2E^{Rh}$ | 0.79 | 610 | -17.6 | 9.1 | -8.5 |
| | $L3E^{Rh}$ | 0.95 | 3745 | -14.1 | 6.7 | -7.4 |
| | $R5E^{Rh}$ | 1.1 | 1740 | -19.1 | 11.2 | -7.9 |
| | $N13D^{Rh}$ | 0.82 | 238 | -19.8 | 10.9 | -8.9 |
| Hck* | $Rh_2(OAc)_4$ | 1.0 (fixed) | 89000 | -4.5 | -1.0 | -5.5 |
| | $S2E^{Rh}$ | 0.95 | 26 | -17.2 | 6.9 | -10.4 |
| | $L3E^{Rh}$ | 0.89 | 22 | -15.3 | 4.9 | -10.4 |
| | $R6E^{Rh}$ | 0.89 | 51 | -10.5 | 0.6 | -9.9 |
| | $P12D^{Rh}$ | 1.0 | 2315 | -9.7 | 2.0 | -7.7 |
| | $P13D^{Rh}$ | 1.0 | 544 | -12.6 | 3.7 | -8.9 |
| Lck | $S2E^{Rh}$ | 1.1 | 481 | -11.2 | 2.6 | -8.6 |
| | $R6E^{Rh}$ | 1.0 | 3788 | -8.8 | 1.4 | -7.4 |
| | $N12D^{Rh}$ | 0.97 | 79 | -12.1 | 2.4 | -9.7 |
| | $N13D^{Rh}$ | 1.1 | 239 | -9.6 | 0.5 | -9.0 |
| Src | $S2E^{Rh}$ | 1.1 | 327 | -16.0 | 7.2 | -8.8 |
| Abl | $S2E^{Rh}$ | 1.5 | 5747 | -17.8 | 10.6 | -7.1 |
| | $N13D^{Rh}$ | 1.5 | 8850 | -12.2 | 5.3 | -6.9 |
| | p40-$A1E^{Rh}$ | 1.0 | 22 | -19.4 | 8.9 | -10.5 |
| | p40-$Y4E^{Rh}$ | 1.2 | 7194 | -12.3 | 5.3 | -7.0 |

* The binding isotherms for metallopeptide interactions with Hck were fitted using a two-sets-of-sites model with the stoichiometry for the second set of sites fixed to 2.0. The second set of binding sites was used to account for the weak, but not negligable, binding of the peptide to another surface of Hck SH3 domain, presumably through non-specific interactions of the dirhodium tetraacetate group. The thermodynamics for the high affinity specific binding site interaction are provided in this table.

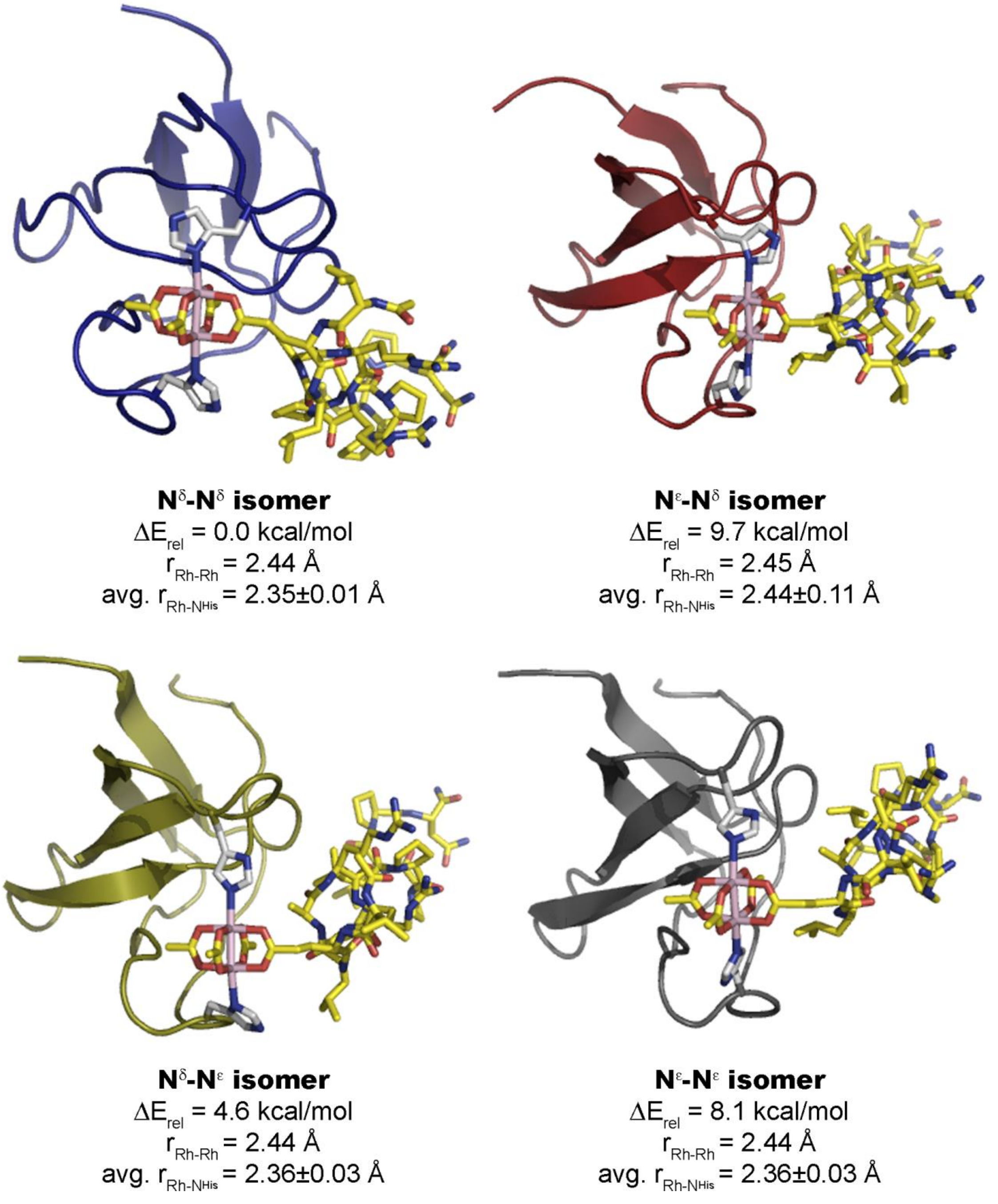


**Figure S16.** Illustrations of QM/MM optimized geometries of **Lyn-Rh$^{S2E}$** isomers with relative total energy and relevant structural metrics. Isomer labels reflect the coordinated nitrogen of the histidine residue with the first being that of His78 followed by His96 (below and above dirhodium, respectively).

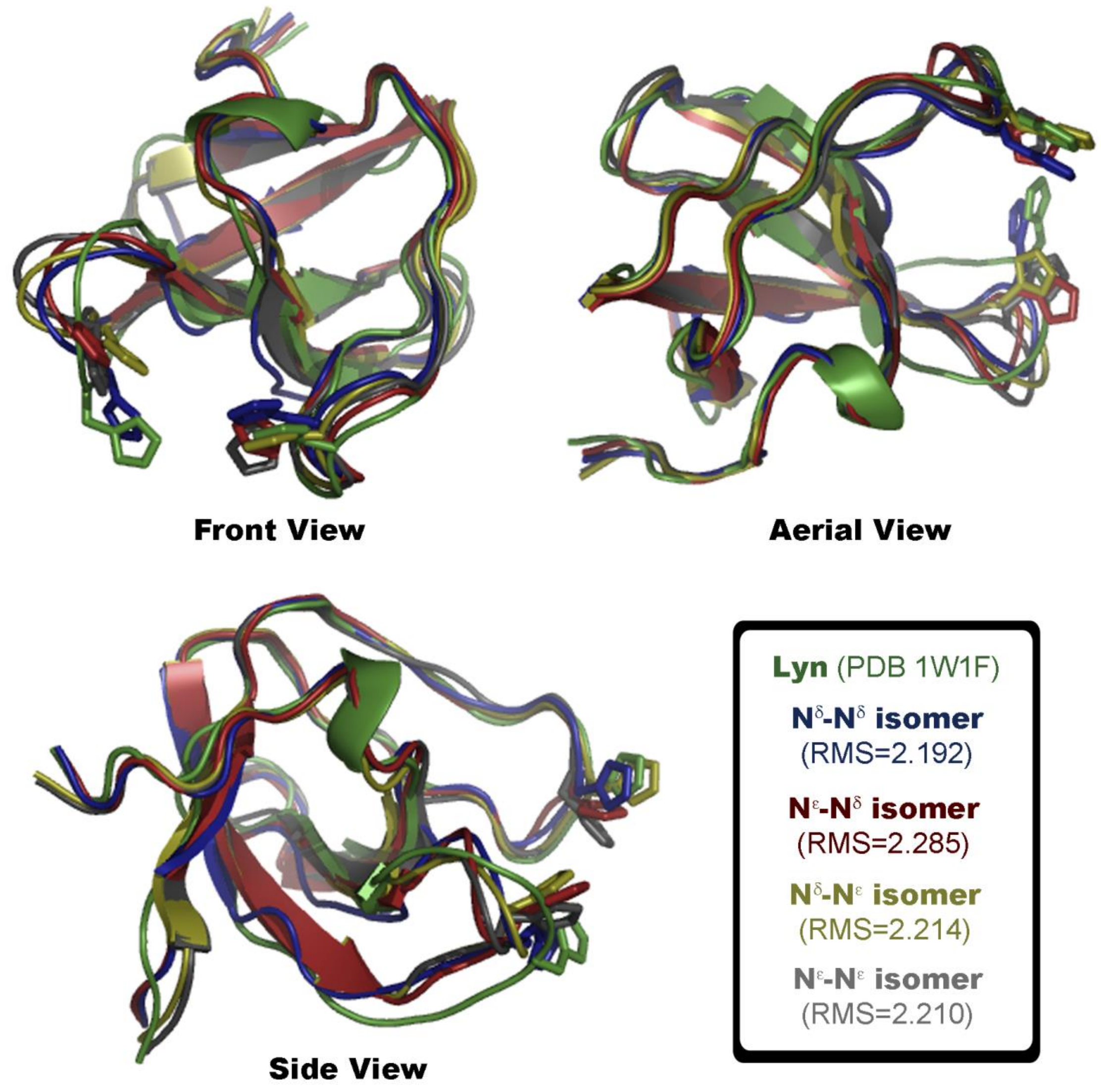


**Figure S17.** Illustrations of QM/MM-optimized **Lyn-Rh$^{S2E}$** isomers overlaid on the experimentally determined structure of Lyn-SH3 domain. **Rh$^{S2E}$** and histidine protons have been removed from the illustration for clarity. Isomer colors correspond to those used in Figure S-1. Visual inspection suggests that significant His78 displacement occurs for all isomers upon dirhodium binding whereas His96 displacement is largely minimized.

**References**

[1] Wellings, D. A.; Atherton, E. In Solid-Phase Peptide Synthesis 1997; Vol. 289, p 44-67.

[2] Wellings, D. A.; Atherton, E. Standard Fmoc protocols. In Methods Enzymol 1997; Vol. 289, p 44-67.

[3] T. Wiseman, S. Williston, J. F. Brandts and L. N. Lin, *Anal. Biochem.*, 1989, **179**, 131-137.

[4] Zhen, Z.; Vohidov, F.; Coughlin, J. M.; Stagg, L. J.; Arold, S. T.; Ladbury, J. E.; Ball, Z. T. *J. Am. Chem. Soc.* **2012**, *134*, 10138-10145.

[5] Vohidov, F.; Coughlin, J. M.; Ball, Z. T., *Angew. Chem. Int. Ed.* **2015**, doi: 10.1002/anie.201411745.

[6] Sivakumar, K.; Xie, F.; Cash, B. M.; Long, S.; Barnhill, H. N.; Wang, Q. Org. Lett. **2004**, *6*, 4603.

[7] Hahn, W. C.; Garrway, L. A.; et al. *Nature* **2010**, *468*, 968–972.

[8] Frisch, M. J.; Trucks, G. W.; Schlegel, H. B.; Scuseria, G. E.; Robb, M. A.; Cheeseman, J. R.; MontgomeryJr., J. A.; Vreven, T.; Kudin, K. N.; Burant, J. C.; Millam, J. M.; Iyengar, S. S.; Tomasi, J.; Barone, V.; Mennucci, B.; Cossi, M.; Scalmani, G.; Rega, N.; Petersson, G. A.; Nakatsuji, H.; Hada, M.; Ehara, M.; K. Toyota; Fukuda, R.; Hasegawa, J.; Ishida, M.; Nakajima, T.; Honda, Y.; Kitao, O.; Nakai, H.; Klene, M.; Li, X.; Knox, J. E.; Hratchian, H. P.; Cross, J. B.; Bakken, V.; Adamo, C.; Jaramillo, J.; Gomperts, R.; Stratmann, R. E.; Yazyev, O.; Austin, A. J.; Cammi, R.; Pomelli, C.; Ochterski, J. W.; Ayala, P. Y.; Morokuma, K.; Voth, G. A.; Salvador, P.; Dannenberg, J. J.; Zakrzewski, V. G.; Dapprich, S.; Daniels, A. D.; Strain, M. C.; Farkas, O.; Malick, D. K.; Rabuck, A. D.; Raghavachari, K.; Foresman, J. B.; Ortiz, J. V.; Cui, Q.; Baboul, A. G.; Clifford, S.; Cioslowski, J.; Stefanov, B. B.; Liu, G.; Liashenko, A.; Piskorz, P.; Komaromi, I.; Martin, R. L.; Fox, D. J.; Keith, T.; Al-Laham, M. A.; Peng, C. Y.; Nanayakkara, A.; Challacombe, M.; Gill, P. M. W.; Johnson, B.; Chen, W.; Wong, M. W.; Gonzalez, C.; Pople, J. A.; Gaussian, Inc.: Wallingford CT, 2004.

[9] (a) Lee, C.; Yang, W.; Parr, R. G. *Phys. Rev. B: Condens. Matter* **1988**, *37*, 785. (b) Becke, A. D. *J. Chem. Phys.* **1993**, *98*, 1372-1377.

[10] (a) Feller, D., J. Comp. Chem., 17(13), 1571-1586, 1996. (b) Schuchardt, K. L.; Didier, B. T.; Elsethagen, T.; Sun, L.; Gurumoorthi, V.; Chase, J.; Li, J.; Windus, T. L. *J. Chem. Inf. Model.* **2007**, *47*, 1045-1052.

[11] Viossat, P.B.; Dung, N.-H.; Rober, F.; Lancelot, J. C.; Robba, M. Acta Crystallogr. **1991**, *C47*, 2550.

[12] The PyMOL Molecular Graphics System, Version 1.5.0.4 Schrödinger, LLC.
